# Supplementary material for: Citrobacter rodentium Relies on Commensals for Colonization of the Colonic Mucosa
Source: Cell Rep. 2017 Dec 19;21(12):3381–9. doi: 10.1016/j.celrep.2017.11.086 (PMC5746604; doi:10.1016/j.celrep.2017.11.086)
Supplement: Document S1. Supplemental Experimental Procedures, Figures S1 and S2, and Tables S1 and S2 [file mmc1.pdf]

Cell Reports, Volume *21*

Supplemental Information

Citrobacter rodentium Relies on Commensals for Colonization of the Colonic Mucosa

Caroline Mullineaux-Sanders, James W. Collins, David Ruano-Gallego, Maayan Levy, Meirav Pevsner-Fischer, Izabela T. Glegola-Madejska, Agnes M. Sågfors, Joshua L.C. Wong, Eran Elinav, Valerie F. Crepin, and Gad Frankel

Supplemental Figures

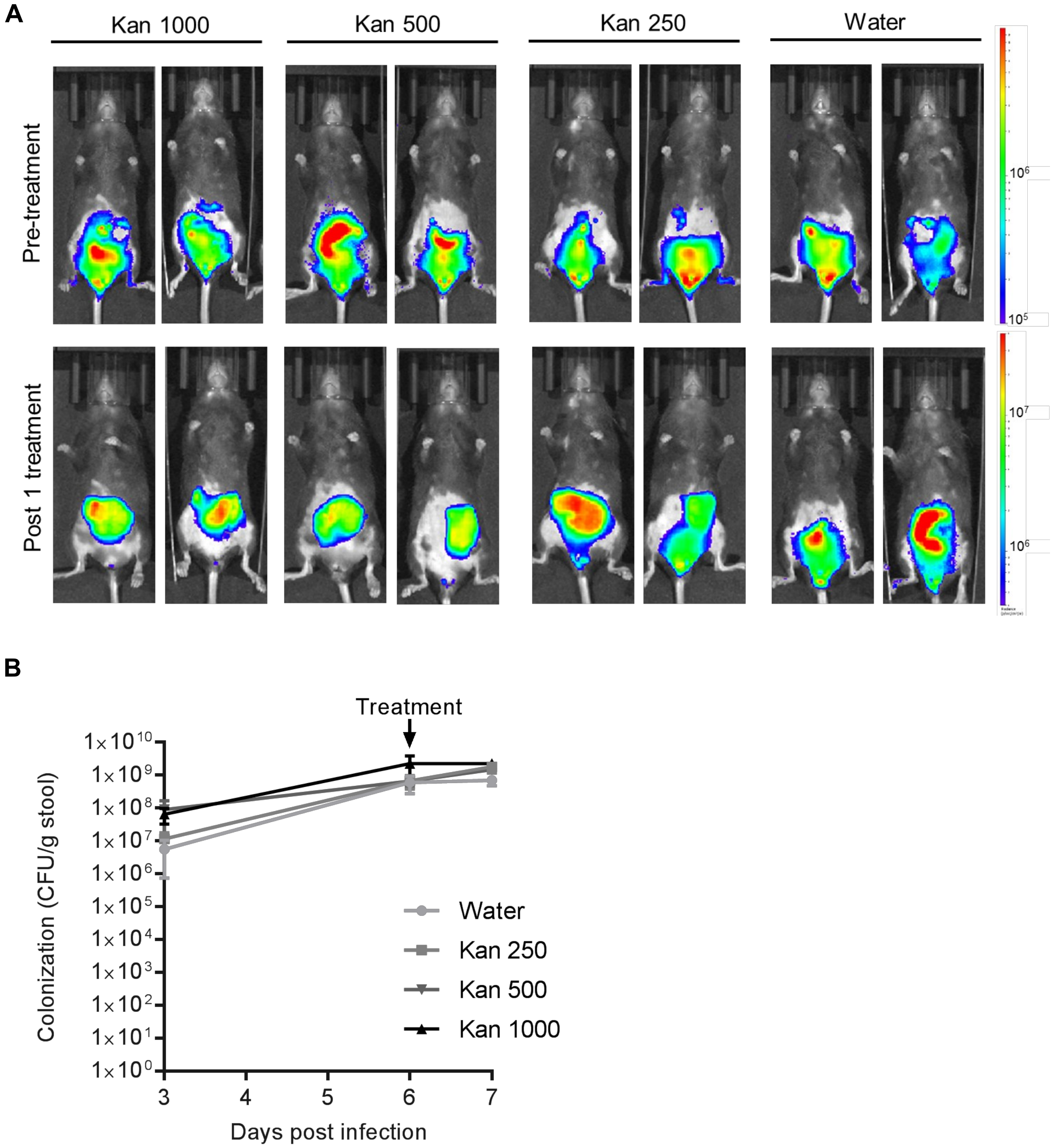

Figure S1. Characterization of the dose-dependent response to Kan treatment; related to Fig. 1. A) *In vivo* BLI of *C. rodentium* from mice before, and 24 h following, a single treatment with 1000 mg/kg, 500 mg/kg, 250 mg/kg Kan or a water control, as indicated. **B)** Quantification of *C. rodentium* CFUs in stools of mice treated as for Fig. S1A. Mean values ± SEM, n=5 mice.

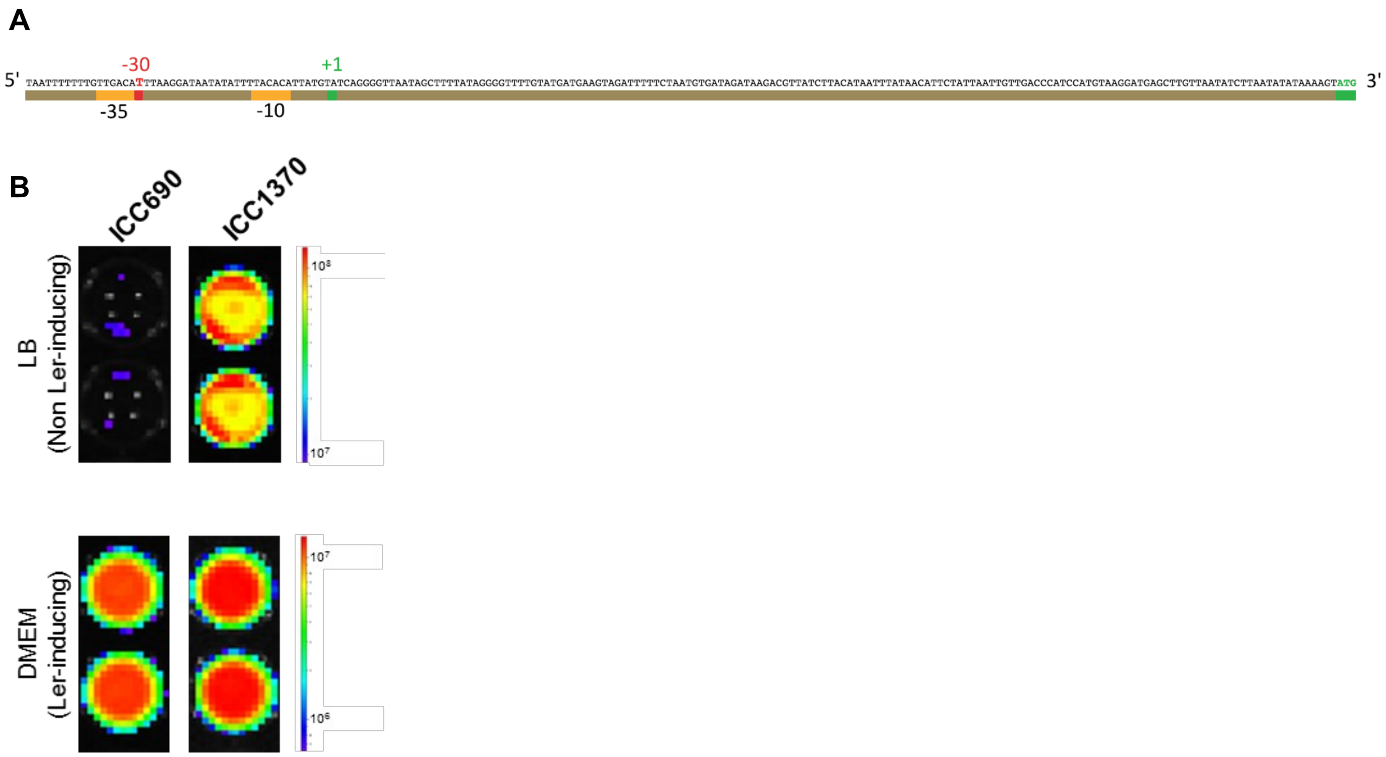

Figure S2. ICC690 and ICC1370; related to Fig. 2C-D and Fig. 5F. A) Sequence of the *ler* promoter; +1=start of transcription; ATG=start of translation. Deletion of the base pair at -30 (shown in red) renders the promoter constitutive. **B)** ICC690 is non-BL when grown under non Ler-inducing conditions (LB); ICC1370 remains BL; ICC690 and ICC1370 are BL when grown under Ler-inducing conditions (DMEM).

Table S1. Strains and Plasmids used in this study; related to Experimental Procedures.

Name	Description	Reference
**Strains**		
E. coli CC118-λpir	Δ(*ara-leu*) *araD* Δ*lacX74 galE galK phoA20 thi- rpsE rpoB argE(Am) recA1, λpir*	De Lorenzo *et al.*, 1990
E. coli 1047 pRK2013	Helper strain for conjugation, KanR, *oriColE1 RK2-Mob+ RK2-Tra+*	Figurski and Helinski, 1979
ICC169	Wild-type *C. rodentium*, O152 serotype, NalR	Wiles *et al.*, 2004
ICC180	Luminescent ICC169 derivative, NalR, KanR	Wiles *et al.*, 2004
ICC690	*C. rodentium* Pler-dependent luminescent strain, NalR, KanR	This study
ICC1370	*C. rodentium* constitutive-Pler luminescent strain, NalR, KanR	This study
ICC1410	ICC180 derivative, Δ*grlR*::CmR; NalR, KanR	This study
**Plasmids**		
pGEmatP2lux	Plasmid harboring the *luxCDABE* operon of *Photorabdus luminescens*	Piñero-Lambea *et al.*, 2014
pACYC184-XylE	pACYC184 derivate; *xylE* homology regions of *C. rodentium*	Girard *et al.*, 2009
pACYC184-XylE-Pler-lux	pACYC184-XylE derivative; *lux* operon under the *C. rodentium ler* promoter	This study
pACYC184-XylE-Pler*-lux	pACYC184-XylE derivative; *lux* operon under *C. rodentium* constitutive *ler* promoter	This study
pICC618	Plasmid encoding the chloramphenicol resistance *cat* cassette	Wong *et al.*, 2012
pSEVA612S	GmR; R6K ori, oriT, I-SceI restriction sites flanking multicloning site	Martínez-García *et al.*, 2014
pSEVAΔ*grlR*-Cm	pSEVA612S derivative; *grlR* homology regions of *C. rodentium* flanking the *cat* cassette	This study
pACBSR	SpR/SmR; p15A ori, PBAD, I-SceI endonuclease and λ-red genes	Ruano-Gallego *et al.*, 2015

Table S2. Primers used in this study; related to Experimental Procedures.

Name	Sequence (5'-3')
5-XbaI-Pler	TAATCTAGAGCTTTGCCAACTAGCTAAATC
3-HindIII-Pler	GACAAGCTTACTTTTATATATTAAGATATTAACAAGC
5-HindIII-lux	AATAGATTCATTCTAGAAAGC
3-SphI-lux	ATTAGCATGCTCAACTATCAAACGCTTCGGTTAAG
5-pler*-GB2	TGATACATAATGTGTAAAATATATTATCCTTAATGTCAACAAAAAAATTATACC
3-pler*-GB2	ACATTAAGGATAATATATTTTACACATTATGTATCAGGGGTTAATAGCTTTTATAG
5-HR-XylE-UP	CTATTGCAGCAACCGCTTTGGCC
3-HR-XylE-DO	AAGTAGTTTGCCAGCCACTGGGC
XylE-UP-gen-check	CAAAATTTAAGTGAATCCGCCGCCAACTCG
3-luxC-seq	TGGGACAAATACAAGGAACTTATCTTCTTC
grlR-gen-check-UP	TTATTTGCATTATCCCTATATCATAGGTTCC
grlR-gen-check-DO	GTCCCACAATACCATTACCAACTCGTAAGG
5-SalI-HR-GrlR	ATCAGTCGACATGGAATCTAAAAATAGTGAC
3-SalI-HR-GrlR	ATCAGTCGACTTTCATGCTAACCTCACTCC

Supplemental Experimental Procedures

Bacterial Strains. The bacterial strains and plasmids used in this study are listed in Table S1. Bacteria were grown at 37ºC on Lysogeny broth (LB) agar (Merck) plates (1.5% w/v), in liquid LB medium (Merck), Dulbecco's modified Eagle's medium (DMEM, Sigma), or M9 minimal medium. When required, antibiotics were added at the following concentrations: chloramphenicol (Cm) 30 μg/ml; kanamycin (Kan) 50 μg/ml; gentamicin (Gm) 10 μg/ml; streptomycin (Sm) 50 μg/ml, nalidixic acid (Nal) 50 μg/ml. We confirmed that ICC180 is sensitive to ciprofloxacin (Cip; <0.25 μg/ml) and resistant to Kan, metronidazole (Met) and vancomycin (Van) (>128 μg/ml).

Lamda Red chromosomal mutations (ICC690 and ICC1370). Primers (Sigma) used in this study are listed in Table S2. The *ler* promoter (Pler) was amplified from *C. rodentium* genomic DNA using primers 5-XbaI-Pler and 3-HindIII-Pler and cloned into pACYC184-XylE. The *lux* operon from *Photorhabdus luminescens* was amplified by PCR from pGEmatP2lux (Piñero-Lambea *et al.*, 2014) with primers 5-HindIII-lux and 3-SphI-lux and inserted in the pCR-BluntII-TOPO plasmid (Zero Blunt TOPO PCR Cloning Kit, Life Technologies). The *lux* operon was subsequently inserted into pACYC184-XylE-Pler to obtain pACYC184-XylE-Pler-lux. To perform the point mutation in the *ler* promoter (Pler*), pACYC184-XylE-Pler-lux was reverse amplified with primers 5-pler*-GB2 and 3-pler*-GB2 and ligated using a Gibson assembly kit (New England Biolabs). To obtain linear products for transformation into ICC169, plasmids pACYC184-XylE-Pler-lux and pACYC184-XylE-Pler*-lux were reverse amplified by PCR using primers 5-HR-XylE-UP and 3-HR-XylE-DO. The PCR products were transformed into electrocompetent ICC169 harbouring pACBSR, previously induced with 0.4% (w/v) L-arabinose (Sigma). Bacteria were incubated for 4 h at 37ºC and Cm-resistant conjugants selected. Integration at the correct genomic location was confirmed by PCR analysis and sequencing using primers XylE-UP-gen-check and 3-luxC-seq. After several passages in liquid LB to remove pACBSR from the strain, bacteria sensitive to Sm were selected.

pSEVA chromosomal mutations (ICC1410). Homology regions flanking the *grlR* gene were synthesized (GenArt, Thermo) and cloned into the suicide vector pSEVA612S (Martínez-García *et al.*, 2014). The construct was then reverse amplified using primers 5-SalI-HR-GrlR and 3-SalI-HR-GrlR and ligated with a Cm resistance cassette digested from pICC618 using SalI. The resulting plasmid pSEVAΔ*grlR*-Cm was conjugated into *C. rodentium* ICC180. Briefly, 20 µl helper *E. coli* 1047 pRK2013 was incubated for 2h at 37°C with 20 µl of the donor strain (*E. coli* CC118-λpir pSEVAΔ*grlR*) on LB agar. Next, 40 µl of the receiver strain (*C. rodentium* ICC180 with pACBSR) was added and the plate incubated for 4 h at 37°C. Conjugants were selected on LB

agar supplemented with Gm and Sm. Individual colonies were grown in LB supplemented with Sm and 0.4% (w/v) L-arabinose (Sigma) for 8 h to induce expression of the I-SceI endonuclease from pACBSR, and plated on Cm plates. Genomic deletion of Δ*grlR* was verified by PCR using primers grlR-gen-check-UP and grlR-gen-check-DO. The strains were passaged several times in liquid LB to remove pACBSR and bacteria sensitive to Sm were selected.

T3SS secretion assay. For the analysis of the T3 secreted proteins, ICC1410 and ICC169 were grown in LB at 37^oC, 200 rpm (non-LEE inducing conditions). To analyze the secretion of T3SS components, culture supernatants were isolated from 1 ml aliquots of induced cultures by centrifugation (20000 g, 5 min). Supernatants were subsequently chilled on ice and incubated for 60 min with trichloroacetic acid (TCA 20% w/v; Sigma) for precipitation. After cold centrifugation (20000 g, 15 min), TCA-precipitated protein pellets were rinsed with cold acetone (-20^oC) and resuspended in 30 µl of Sodium Dodecyl Sulfate–Polyacrylamide gel electrophoresis (SDS-PAGE) sample buffer.

Western blots. SDS-PAGE and Western blot were performed following standard methods using the Miniprotean III system (Bio-Rad). Proteins separated by SDS-PAGE were either subjected to Coomassie Blue R-250 (Sigma) staining or Western blot. For the latter, the proteins were transferred to a polyvinylidene difluoride membrane (PVDF, GE Healthcare) using semi-dry electrophoresis (Bio-Rad). Membranes were incubated with rabbit anti-EspA (1:5000) or anti-EspB (1:5000) antibodies followed by secondary goat anti-rabbit antibody conjugated to HRP (1:500, Jackson ImmunoResearch). Membranes were developed by chemiluminescence using the EZ-ECL kit (Biological Industries) and observed in a Fuji LAS 3000 Imager (Fujifilm).

Cell culture and infection. Swiss 3T3 and HeLa (ATCC) cell lines were cultured and seeded onto glass coverslips in 24-well plates and infected with *C. rodentium* as previously described (Girard *et al.*, 2009). For ICC180 and ICC1410 infections, cultures grown for 8 hours in LB (37^oC, 200 rpm) were diluted 1/100 in DMEM and grown overnight at 37^oC, 5% CO_2. 100 µl of overnight culture was added to each well. Plates were incubated at 37^oC, 5% CO_2 for 3 hours. Cells were washed in PBS and fixed for 20 min in 4% paraformaldehyde (Collins *et al.*, 2014b). For infections with *C. rodentium* isolated from infected mice, fresh fecal samples taken from donor mice were diluted 1/10 in PBS, homogenized and then 20 µl of the supernatant was immediately added to the wells (Bishop *et al.*, 2007). Control wells were infected with 100 µl of an overnight culture, prepared as described above. Plates were centrifuged at 1000 rpm for 5 min at room temperature, and were incubated at 37°C in 5% CO_2 for 5 h, then washed and fixed as described above.

Colonic crypt measurements. 0.5 cm distal colon was harvested, fixed in formalin, sectioned and stained with hematoxylin and eosin as previously described (Crepin *et al.,* 2016). Crypt hyperplasia was determined by measuring the lengths of at least 20 well-oriented crypts from each section, from all of the mice per treatment group. All histological sections were evaluated blindly.

Indirect immunofluorescence staining. Fixed cells were permeabilized in 0.1% Triton X100 (Sigma) for 10 min at room temperature. For tissue sections, indirect immunofluorescence was performed following heat induced epitope retrieval of formalin-fixed paraffin embedded sections as previously described (Collins *et al.*, 2014b). Samples were treated with primary rabbit polyclonal anti-*C. rodentium* (1:50 for tissue sections, 1:200 for fixed cells; Sigma) and mouse anti-E-Cadherin antibodies (1:50; CD324, BD Biosciences). Samples were subsequently treated with secondary antibodies: Donkey anti-rabbit Alexa Flour 488, donkey anti-mouse Alexa Flour 488 or donkey anti-rabbit Alexa Flour 555 (all 1:200; Jackson Immunoresearch). Actin was counter-stained with Phalloidin (Stratech) and DNA was counter-stained with Hoescht 33342 or DAPI. Images were acquired using a Zeiss AxioVision Z3 microscope, and processed using Zen 2.3 (Blue Version) (Carl Zeiss MicroImaging GmbH, Germany).

Supplemental References

Bishop, A.L., Wiles, S., Dougan, G., and Frankel, G. (2007). Cell attachment properties and infectivity of host-adapted and environmentally adapted *Citrobacter rodentium*. Microb. Infect. *9*, 1316-1324.

Collins, J.W., Chervaux, C., Raymond, B., Derrien, M., Brazeilles, R., Kosta, A., Chambaud, I., Crepin, V.F., and Frankel, G. (2014b). Fermented dairy products modulate *C. rodentium* induced colonic hyperplasia. J. Infect. Dis. *210*, 1029-1041.

De Lorenzo, V., Herrero, M., Jakubzik, U., and Timmis, K.N. (1990). Mini-Tn5 transposon derivatives for insertion mutagenesis, promoter probing, and chromosomal insertion of cloned DNA in gram-negative eubacteria. J. Bacteriol. *172*, 6568-6572.

Figurski, D.H., and Helinski, D.R. (1979). Replication of an origin-containing derivative of plasmid RK2 dependent on a plasmid function provided in trans. Proc. Natl. Acad. Sci. U.S.A. *76*, 1648-1652.

Girard, F., Crepin, V.F., and Frankel, G. (2009). Modelling of infection by enteropathogenic *Escherichia coli* strains in lineages 2 and 4 *ex vivo* and *in vivo* by using *Citrobacter rodentium* expressing TccP. Infect. Immun. *77*, 1304-1314.

Martínez-García, E., Aparicio, T., Goñi-Moreno, A., Fraile, S., and De Lorenzo, V. (2014). SEVA 2.0: an update of the Standard European Vector Architecture for de-/re-construction of bacterial functionalities. Nucleic Acids Res. *43,* D1183–D1189.

Piñero-Lambea, C., Bodelón, G., Fernández-Periáñez, R., Cuesta, A.M., Álvarez-Vallina, L., and Fernández, L.A.n. (2014). Programming controlled adhesion of *E. coli* to target surfaces, cells, and tumors with synthetic adhesins. ACS Synth. Biol. *4*, 463-473.

Ruano-Gallego, D., Álvarez, B., and Fernández, L.A.n. (2015). Engineering the controlled assembly of filamentous injectisomes in *E. coli* K-12 for protein translocation into mammalian cells. ACS Synth. Biol. *4*, 1030-1041.

Wong, A.R., Raymond, B., Collins, J.W., Crepin, V.F., and Frankel, G. (2012). The enteropathogenic *E. coli* effector EspH promotes actin pedestal formation and elongation via WASP‐interacting protein (WIP). Cell Microbiol. *14*, 1051-1070.
