# Supplementary material for: Citrobacter rodentium Relies on Commensals for Colonization of the Colonic Mucosa
Source: Cell Rep. 2017 Dec 19;21(12):3381–9. doi: 10.1016/j.celrep.2017.11.086 (PMC5746604; doi:10.1016/j.celrep.2017.11.086)
Supplement: Document S2. Article plus Supplemental Information [file mmc2.pdf]

Report

Cell Reports

Citrobacter rodentium Relies on Commensals for Colonization of the Colonic Mucosa

Graphical Abstract

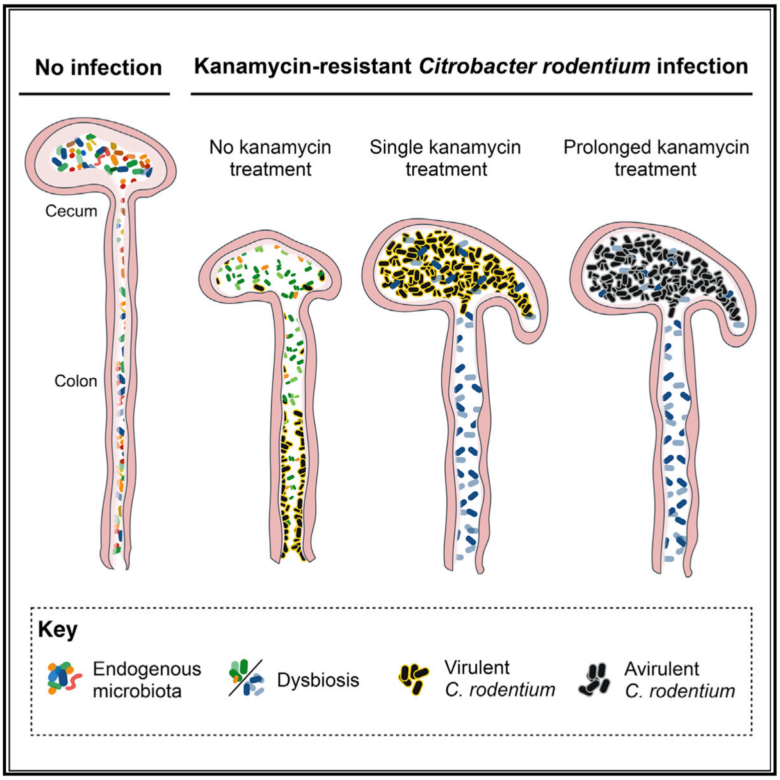

Highlights

- Specific dysbiosis rapidly displaces *Citrobacter rodentium* from the colonic mucosa
- Mucosal exclusion is independent of *C. rodentium* virulence gene expression
- Extended antibiotic treatment causes accumulation of luminal avirulent *C. rodentium*
- *C. rodentium* relies on commensals for survival at the colonic mucosa

Authors

Caroline Mullineaux-Sanders, James W. Collins, David Ruano-Gallego, ..., Eran Elinav, Valerie F. Crepin, Gad Frankel

Correspondence

g.frankel@imperial.ac.uk

In Brief

A/E pathogens intimately adhere to the gut mucosa. Mullineaux-Sanders et al. demonstrate that inducing specific dysbiosis at the peak of murine infection with *Citrobacter rodentium* prevents mucosal colonization. This occurs via a mechanism independent of virulence gene expression modulation, indicating that enteric pathogens may rely on commensals for effective infection.

Mullineaux-Sanders et al., 2017, Cell Reports *21*, 3381–3389
December 19, 2017 © 2017 The Author(s).
https://doi.org/10.1016/j.celrep.2017.11.086

CellPress

Cell Reports
Report

OPEN ACCESS
CellPress

Citrobacter rodentium Relies on Commensals for Colonization of the Colonic Mucosa

Caroline Mullineaux-Sanders,[1] James W. Collins,[1] David Ruano-Gallego,[1] Maayan Levy,[2] Meirav Pevsner-Fischer,[2] Izabela T. Glegola-Madejska,[1] Agnes M. Sågfors,[1] Joshua L.C. Wong,[1,3] Eran Elinav,[2] Valerie F. Crepin,[1] and Gad Frankel[1,4,*]
[1]MRC Centre for Molecular Bacteriology and Infection, Department of Life Sciences, Imperial College, London, UK
[2]Department of Immunology, The Weizmann Institute of Science, Rehovot, Israel
[3]Section of Anaesthetics, Pain Medicine and Intensive Care, Department of Surgery and Cancer, Imperial College London, London, UK
[4]Lead Contact
*Correspondence: g.frankel@imperial.ac.uk
https://doi.org/10.1016/j.celrep.2017.11.086

SUMMARY

We investigated the role of commensals at the peak of infection with the colonic mouse pathogen *Citrobacter rodentium.* Bioluminescent and kanamycin (Kan)-resistant *C. rodentium* persisted avirulently in the cecal lumen of mice continuously treated with Kan. A single Kan treatment was sufficient to displace *C. rodentium* from the colonic mucosa, a phenomenon not observed following treatment with vancomycin (Van) or metronidazole (Met). Kan, Van, and Met induce distinct dysbiosis, suggesting *C. rodentium* relies on specific commensals for colonic colonization. Expression of the master virulence regulator *ler* is induced in germ-free mice, yet *C. rodentium* is only seen in the cecal lumen. Moreover, in conventional mice, a single Kan treatment was sufficient to displace *C. rodentium* constitutively expressing Ler from the colonic mucosa. These results show that expression of virulence genes is not sufficient for colonization of the colonic mucosa and that commensals are essential for a physiological infection course.

INTRODUCTION

Antibiotic chemotherapy is often an effective treatment for bacterial infections, leading to a rapid reduction in bacterial burden, morbidity, and mortality. However, unintentional targets of oral antibiotics are commensal bacteria, which provide a protective barrier against pathogens (Kamada et al., 2013; Zhang et al., 2013). Antibiotic-induced dysbiosis increases host susceptibility to bacterial colonization; by pre-treating mice with streptomycin pathogenic and non-pathogenic bacteria, e.g., *Salmonella enterica* serovar Typhimurium and *Escherichia coli*, can colonize the murine gastrointestinal tract, which is usually refractory to these strains (Barthel et al., 2003; Spees et al., 2013). However, little attention has been paid to the consequences of antibiotic treatment during physiological enteric infections, which occur in the context of the endogenous microbiota, and the impact of antibiotic treatment on host physiology (e.g., streptomycin causes cecal enlargement).

Citrobacter rodentium is an extracellular enteric murine pathogen that shares an infection strategy and virulence factors with the human diarrheagenic pathogens enteropathogenic and enterohemorrhagic *E. coli* (EPEC and EHEC) (Collins et al., 2014). In C57BL/6 mice, *C. rodentium* causes a self-limiting infection, without the need for antibiotic pre-treatment, and triggers robust colitis, colonic crypt hyperplasia (CCH), and dysbiosis (Collins et al., 2014). Following oral inoculation, *C. rodentium* colonizes the cecum, where the pathogen adapts to the *in vivo* environment of the gut and from where it spreads to the distal colon and undergoes rapid expansion (Wiles et al., 2004). Colonization of *C. rodentium* plateaus 6 or 7 days post-infection (DPI) and starts to clear 10–12 DPI (Collins et al., 2013; Wiles et al., 2004). *C. rodentium* colonizes the colonic mucosa while forming attaching and effacing (A/E) lesions, which are characterized by effacement of the brush border microvilli underneath attached bacteria (Collins et al., 2014). Infection of cultured cells with *C. rodentium* leads to the formation of actin-rich pedestal-like structures (Collins et al., 2014; Crepin et al., 2010). The ability to form A/E lesions and pedestals is conferred by the locus of enterocyte effacement (LEE) pathogenicity island (McDaniel et al., 1995), which encodes the transcriptional regulators Ler (Mellies et al., 1999), GrlA, and GrlR (Deng et al., 2004); the adhesin intimin; a type III secretion system (T3SS); and effectors (Garmendia et al., 2005; Wong et al., 2011). Mutants in *ler*, in which the LEE is not expressed, are avirulent, yet they can colonize germ-free mice (Kamada et al., 2012).

The gut microbiota is known to have a significant impact on *C. rodentium* disease course. Transplantation of the gut microbiota from C57BL/6 mice to lethally susceptible C3H/HeOuJ mice prevented mortality (Ghosh et al., 2011); *Bacteroides thetaiotaomicron* influences *C. rodentium* virulence gene expression via alteration of the metabolic landscape (Curtis et al., 2014), and gut commensals are necessary for effective clearance of luminal *C. rodentium* following infection (Kamada et al., 2012).

In this study, we used the bioluminescent (BL) and kanamycin (Kan)-resistant *C. rodentium* strain ICC180 and antibiotic treatment to test the effect of disrupting commensal bacteria during the peak of an acute enteric infection.

This is an open access article under the CC BY license (http://creativecommons.org/licenses/by/4.0/).

OPEN ACCESS
CellPress

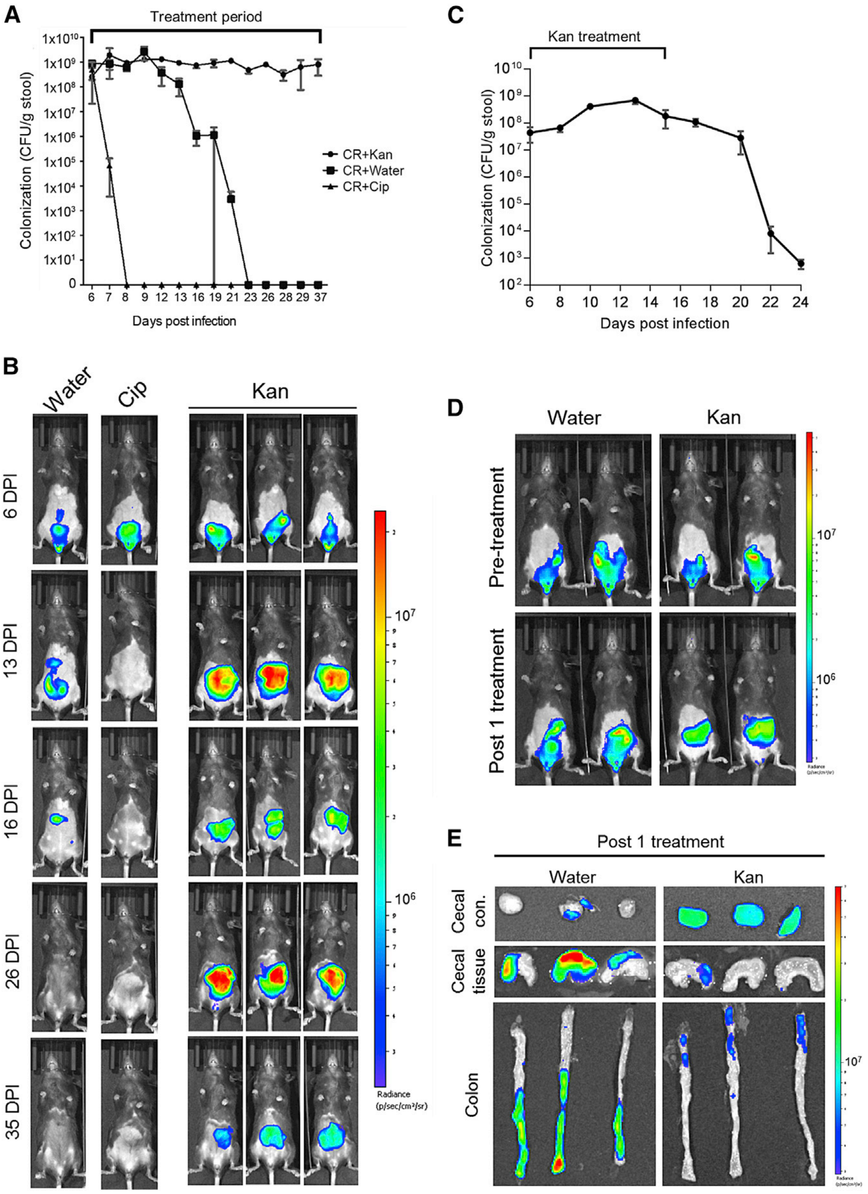

Figure 1. Kan Treatment during *C. rodentium* Infection Triggers AIBP and Prevents Colonic Colonization

Mice infected with *C. rodentium* were treated daily with Kan (1,000 mg/kg), Cip (100 mg/kg), or water from 6 DPI.

(A) Quantification of *C. rodentium* CFUs in stool from 6 to 37 DPI. Mean values ± SEM; n = 5–6 mice.

(B) *In vivo* BLI of *C. rodentium* from representative mice, showing a redistribution of the BL signal from the colon to the cecum.

(C) Quantification of *C. rodentium* CFUs in stool, following discontinuation of Kan treatment at 15 DPI, demonstrating AIBP is a transient state. Mean values ± SEM; n = 3 mice.

(D) *In vivo* BLI of *C. rodentium* from representative mice prior to treatment (at 6 DPI) and one day post-Kan treatment. See also Figure S1.

(E) *Ex vivo* BLI of the cecal tissue and contents and colonic tissue 1 day post a single 1,000 mg/kg Kan treatment (7 DPI) from representative mice. Bright BL signal is seen in the cecal contents whereas little signal is observed on the cecal and colonic mucosa of the Kan-treated mice.

RESULTS

Treating ICC180 Infection with Kan Leads to Bacterial Persistence

We determined the impact of disturbing the microbiota with daily oral treatments of C57BL/6 mice with Kan (1,000 mg/kg/day) during the acute phase of ICC180 infection, from 6 DPI; ICC180 is resistant to >500 μg/mL Kan *in vitro* (not shown). Treatments with ciprofloxacin (Cip) (100 mg/kg/day) and water were used as controls. Enumeration of bacterial shedding and *in vivo* bioluminescent imaging (BLI) revealed that the water-treated group followed typical clearance dynamics and Cip-treated mice cleared *C. rodentium* within 48 hr (Figures 1A and 1B). In contrast, daily oral treatments with Kan resulted in the number of shed *C. rodentium* plateauing at around 10^9 colony-forming units (CFUs)/g of feces for the duration of the study (Figure 1A), a phenomenon we term antibiotic-induced bacterial persistence (AIBP). Stopping the Kan treatment resulted in rapid clearance of the infection (Figure 1C), suggesting the AIBP state is transient. BLI revealed that AIBP was accompanied by redistribution of the BL signal from the colon and cecum prior to Kan treatment to solely the cecum post-treatment (Figure 1B).

Investigating the kinetics of AIBP induction revealed that a single Kan treatment was sufficient to cause redistribution of the BL signal from the colon to the cecum (Figures 1D and 1E). Characterization of the dose-dependent response to Kan showed that, following a single treatment with 500 mg/kg, all mice exhibited cecal BL (Figure S1A). In contrast, following a single treatment with 250 mg/kg, this occurred in only 50% of the mice, whereas in the water-treated controls, BL remained visible in the colon of all animals (Figure S1A); *C. rodentium* shedding was comparable in all groups (Figure S1B). *Ex vivo* BLI showed that, following a single Kan treatment, *C. rodentium* is confined to the cecum, specifically the luminal cecal contents, with no BL signal visible on the distal colon (Figure 1E).

C. rodentium in the AIBP State Is Avirulent

We next examined the infectivity of *C. rodentium* after 20 Kan treatments (26 DPI) by co-housing mice harboring AIBP *C. rodentium* with naive untreated mice; untreated mice infected with *C. rodentium* for 7 days were used as a control. Whereas

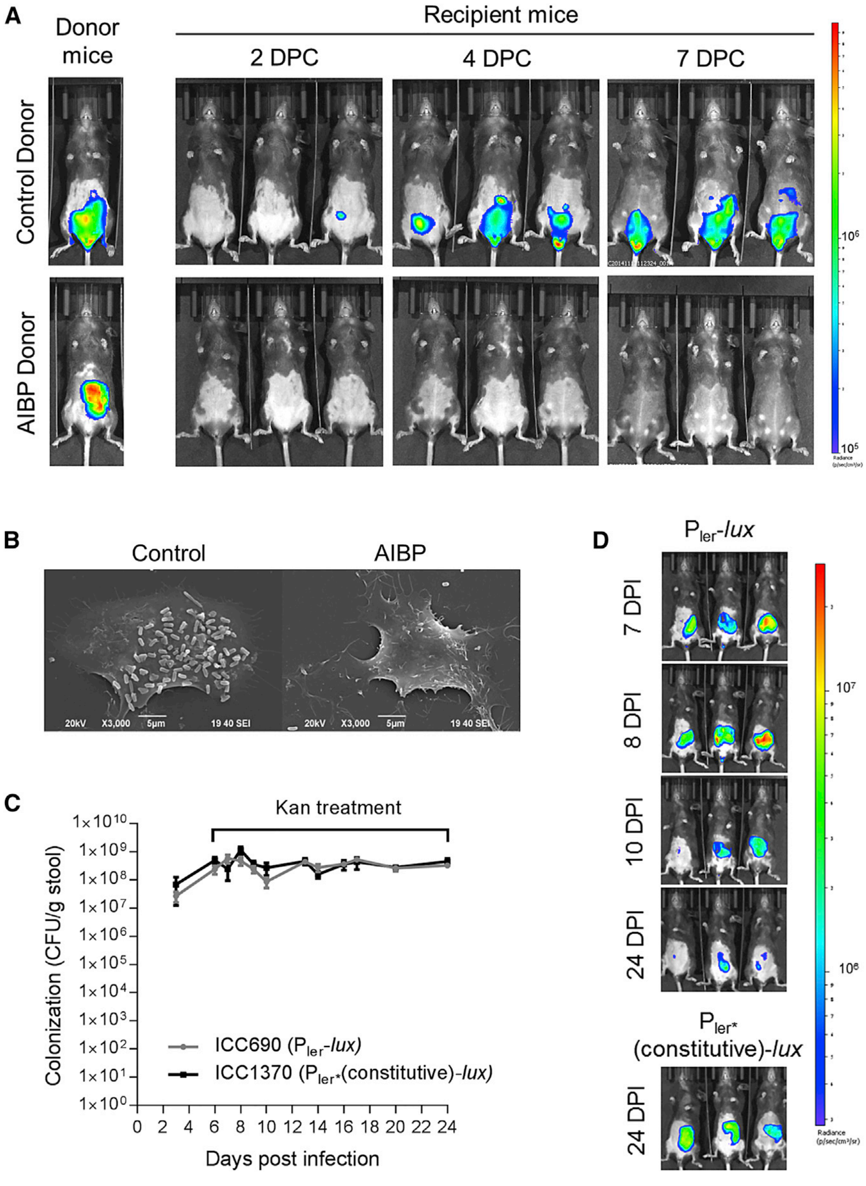

Figure 2. *C. rodentium* in the AIBP State Are Non-infectious

(A) *In vivo* BLI of control and AIBP-donor mice and co-housed naive untreated recipient mice at 2, 4, and 7 days post co-habitation (DPC), showing AIBP *C. rodentium* is not transmissible.

(B) Scanning electron microscopy of mouse fibroblasts infected with *C. rodentium* isolated from feces of control or AIBP mice. Control *C. rodentium*, but not AIBP *C. rodentium*, are able to induce pedestal formation. Images are at a magnification of 3,000×; scale bar, 5 μm.

(C) Quantification of *C. rodentium* CFUs in stools of mice infected with ICC690 (P_{ler}-*lux*) or ICC1370 (P_{ler}*(constitutive)-*lux*) and treated with Kan (500 mg/kg/day). Mean values ± SEM; n = 5 mice.

(D) *In vivo* BLI of representative mice, showing dimming of the ICC690 BL signal over time, demonstrating *ler* downregulation in the AIBP state.

See also Figure S2.

mice co-housed with control animals developed a robust colonic infection by 4 days post-co-habitation, those co-housed with the AIBP donor did not (Figure 2A). Further, AIBP *C. rodentium* taken directly from re-suspended feces was unable to adhere to cultured mouse fibroblasts, whereas *C. rodentium* from control animals readily triggered pedestal formation (Figure 2B). Passage of AIBP *C. rodentium* in lysogeny broth (LB) prior to infection restored pedestal formation (not shown). Taken together, these data suggest a reversible repression of virulence gene expression in AIBP *C. rodentium*.

To directly visualize virulence gene expression in the AIBP state, we infected mice with a reporter *C. rodentium* strain (ICC690), in which a chromosomal *lux* operon is under the control of the *ler* promoter. A strain containing a single base pair deletion in the *ler* promoter (position −30; Figure S2A), rendering the promoter constitutively active (Islam et al., 2011), was used as a control (ICC1370; Figure S2B). Following oral inoculation, both ICC690 and ICC1370 were shed at comparable numbers (Figure 2C). A single dose of Kan resulted in redistribution of the BL of ICC690 and ICC1370 from the colon to the cecum. Whereas the BL signal in ICC690 diminished following 4 daily Kan treatments (10 DPI) and remained dim (Figure 2D), the BL signal remained visible in the cecum of ICC1370-infected mice at 24 DPI (Figure 2D). These results show that expression of *ler*, and by extension expression of the LEE, is downregulated in the AIBP state.

Kan-Induced Disruption of the Microbiota Displaces Colonic *C. rodentium*

Despite colonizing the colonic mucosa at 6 DPI, prior to antibiotic treatment, little to no BL signal was observed on the distal colon following a single dose, suggesting that Kan treatment displaces colonic *C. rodentium* (Figure 1E). To confirm that the lack of BL reflects an absence of mucosal-attached *C. rodentium*, the tissue-associated colonic CFUs of Kan-treated (1,000 mg/kg) mice were determined; water-treated mice were used as a control. Comparable levels of ICC180 were shed in the stool of both groups (Figure 3A); however, whereas significantly more *C. rodentium* were found in the cecal content, significantly fewer ICC180 were present in the distal colon of the Kan-treated, compared to the water-treated, mice (Figure 3B). Immunofluorescence staining of *C. rodentium* further confirmed that little to no *C. rodentium* was present on the colonic mucosa following a single Kan treatment (Figure 3C). As CCH and elevated fecal Lipocalin-2 (LCN-2) are known markers of

OPEN
ACCESS
CellPress

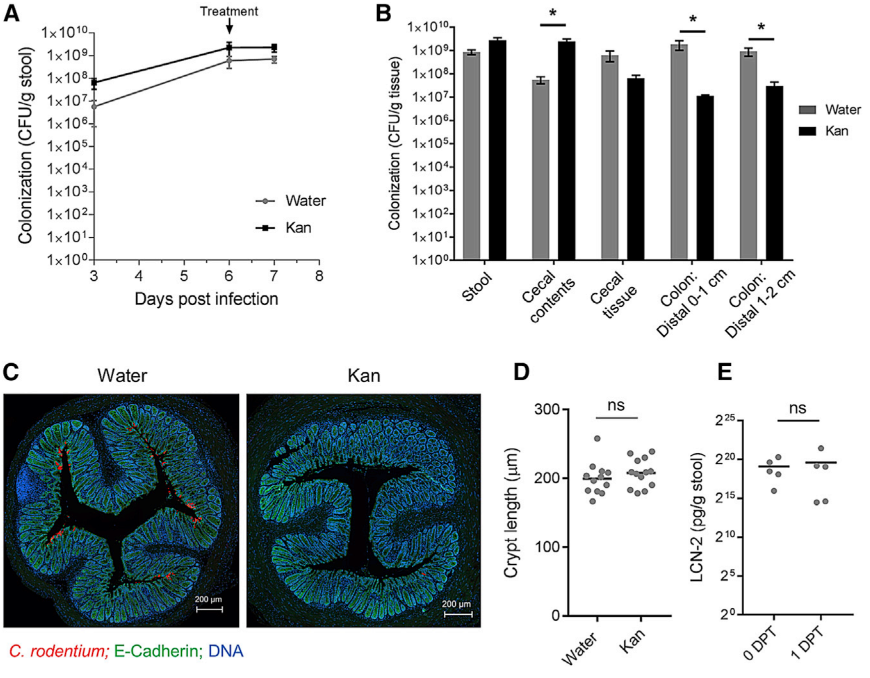

Figure 3. Kan Treatment Displaces *C. rodentium* from the Colonic Mucosa

ICC180-infected mice were given a single Kan (1,000 mg/kg) or water treatment at 6 DPI and harvested 24 hr later.

(A) Quantification of *C. rodentium* CFUs in the stool.

(B) Quantification of tissue-associated *C. rodentium* CFUs, revealing significantly elevated bacterial load in the cecal contents and decreased CFUs in the distal colons of Kan-treated mice. (A) and (B) show mean values ± SEM; n = 4 mice. Significance was determined by an unpaired two-tailed Student's t test.

(C) Indirect immunofluorescence of sections of 0.5 cm distal colon, showing little to no *C. rodentium* present on the Kan-treated colon. *C. rodentium* staining is in red, E-cadherin in green, and DNA in blue. The scale bar represents 200 μm.

(D) Colonic crypt lengths of 0.5-cm distal colon. Each data point represents the mean crypt length of a single mouse. (B and D) Significance was determined by an unpaired two-tailed Student's t test.

(E) Stool LCN-2 concentrations of the same mice at 6 DPI (untreated; 0 DPT) and 1 day following a single Kan treatment (1 DPT). Each dot represents a single mouse. Significance was determined by a paired two-tailed Student's t test. *p < 0.05; ns, not significant.

C. rodentium infection (Collins et al., 2014; unpublished data), we investigated whether these parameters were impacted by a single Kan treatment. No significant differences in CCH or fecal LCN-2 were observed after a single Kan treatment compared to water-treated controls or mice prior to treatment, respectively (Figures 3D and 3E).

Met and Van Treatments Do Not Induce AIBP

To test whether the inability to colonize the colonic mucosa is the result of the specific depletion of Kan-sensitive commensals or a general reduction in microbiotal diversity, we tested the effect of two other classes of antibiotics that *C. rodentium* resists: vancomycin (Van) (50 mg/kg/day) and metronidazole (Met) (100 mg/kg/day). Kan, Van, and Met induced distinct taxonomic changes to the fecal microbiota (Figure 4A) and caused a significant reduction in alpha diversity (Figure 4B). BLI showed that, following Van or Met treatments, ICC180 remained visible on the colonic mucosa (Figure 4C). Therefore, the depletion of commensals by a single Kan treatment, but not Van or Met treatments, displaces *C. rodentium* from the colonic mucosa.

Antibiotic-treated animals have reduced gut motility and display enlarged ceca (Wostmann and Bruckner-Kardoss, 1960). However, measuring the gut transit times of antibiotic-treated mice showed no significant reduction after a single treatment with Kan, Van, or Met compared to water-treated controls (Figure 4D), suggesting this is not related to the inability of *C. rodentium* to colonize the colonic mucosa following Kan treatment.

The Role of Ler Expression in *C. rodentium* Colonic Colonization during Kan Treatment

Members of the gut microbiota have been shown to indirectly modulate EHEC and *C. rodentium* virulence gene expression (Curtis et al., 2014). Therefore, to investigate whether the inability of *C. rodentium* to colonize the colonic mucosa of Kan-treated mice is due to repression of *ler*, we deleted the negative regulator GrlR from ICC180 to generate ICC1410, which constitutively expresses *ler* (Lio and Syu, 2004). ICC1410 hyper-secretes translocators and effectors in non-*ler*-inducing conditions (Figure 5A) and infects cultured cells more robustly than ICC180 (Figure 5B). Following infections of conventional mice, ICC1410 behaved similarly to ICC180 and colonized the colonic mucosa at 6 DPI (Figures 5C and 5D). Importantly, following a single Kan dose, the BL signal was found exclusively in the cecal content (Figure 5D), demonstrating *ler* downregulation does not account for the inability of ICC180 to colonize the colonic mucosa following Kan treatment.

C. rodentium Colonizes the Cecal Lumen of Germ-free Mice

As disruption of the microbiota affects the ability of *C. rodentium* to colonize the colonic mucosa, we next investigated the outcome of infection of germ-free mice with ICC690 and ICC1370. CFU enumeration showed that, similarly to Kan-treated mice and consistent with previous data (Kamada et al., 2012), germ-free mice persistently shed *C. rodentium* at high titers (Figure 5E). Whereas the BL signal in ICC690-infected mice was dim 2 DPI and bright 8 DPI, a diminishing signal was seen at 12 and 14 DPI (Figure 5F). A bright signal from the control ICC1370 was seen at 14 DPI (Figure 5F), with similar CFUs to ICC690 shed in the stool (Figure 5E). These results suggest that cues from the host can upregulate *ler* gene expression during early *C. rodentium* infection *in vivo*. Importantly, despite strong *ler* expression at 8 DPI, *ex vivo* BLI and CFU enumeration revealed *C. rodentium* primarily reside in the cecal content

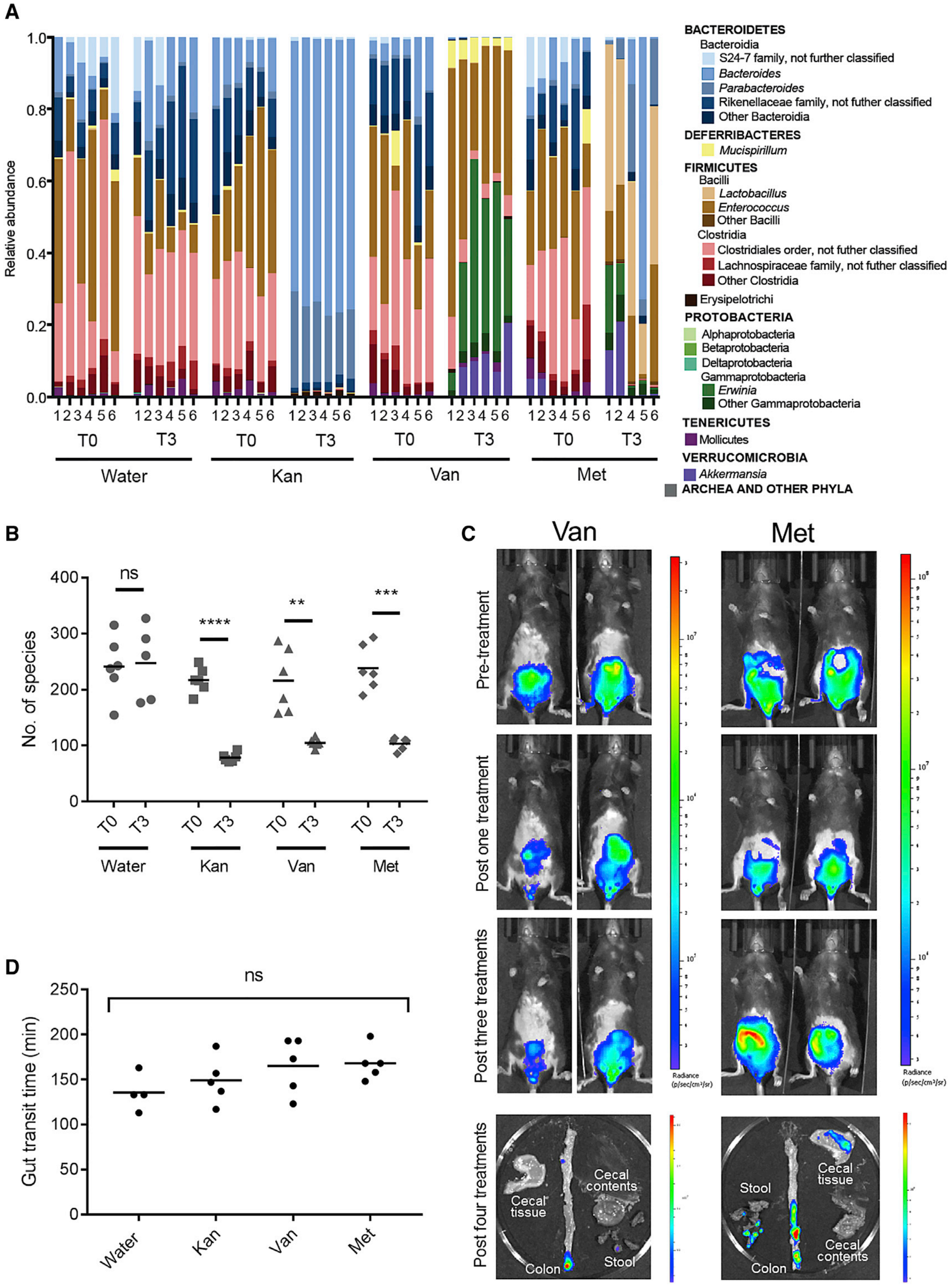

Figure 4. Vancomycin and Metronidazole Do Not Prohibit Colonic Colonization

(A and B) 16S RNA sequencing analysis of stools collected from uninfected mice prior to antibiotic treatment (T0) and 24 hr after 3 daily treatments of Kan (1,000 mg/kg/day), Van, Met, or a water control (T3). (A) The relative abundance of taxa is shown. (B) Alpha diversity of the species in each of the treatment groups after T0 and T3 is shown. Significance was determined by a paired two-tailed Student's t test.

(C) *In vivo* and *ex vivo* BLI of ICC180-infected mice treated with Van or Met, showing *C. rodentium* remain associated with the colonic mucosa.

(D) Total gut transit time of uninfected mice 24 hr following a single Kan (1,000 mg/kg), Van, Met, or water treatment, showing no significant differences in gut motility. Significance was determined by a one-way ANOVA with Tukey's multiple comparison post-test. $^{**}p < 0.01$; $^{***}p < 0.001$; $^{****}p < 0.0001$.

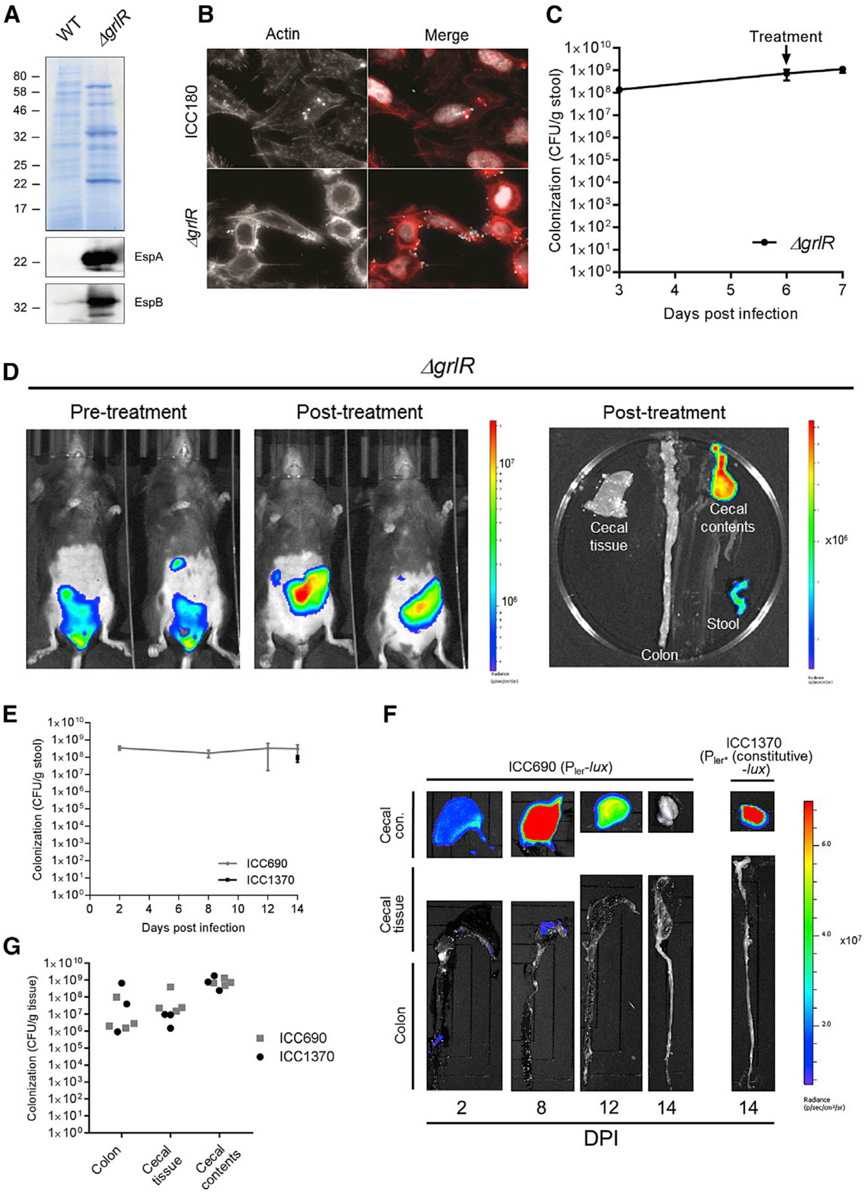

Figure 5. Expression of Virulence Genes Is Not Sufficient for Colonic Colonization

(A) Coomassie staining and the corresponding western blot (EspA and EspB) of bacterial culture supernatants, demonstrating constitutive T3S in ICC1410.

(B) Indirect immuno-fluorescence staining of representative HeLa cells infected with ICC180 or ICC1410. In right-hand panels, actin staining is in red, *C. rodentium* in cyan, and DNA in white.

(C and D) Mice infected with *C. rodentium ΔgrlR* (ICC1410) were given a single Kan (1,000 mg/kg) treatment at 6 DPI. (C) Quantification of *C. rodentium* CFUs in the stool is shown. Mean ± SEM; n = 3 mice. (D) *In vivo* BLI at 6 DPI (pre-treatment) and 24 hr post-Kan treatment and corresponding *ex vivo* BLI of excised organs is shown, demonstrating bright BL in the cecal content and diminished mucosal BL signal following infection with *C. rodentium* constitutively expressing the LEE.

(E–G) Germ–free C57BL/6 mice were infected with the P_{ler}-*lux* reporter stain ICC690 or the P_{ler}*(constitutive)-*lux* control strain ICC1370. (E) Quantification of *C. rodentium* CFUs in the stool is shown. Mean ± SEM; n = 3–5 mice. (F) Representative *ex vivo* BLI of excised organs is shown, demonstrating confinement of the BL signal to the cecal contents, despite strong *ler* expression at 8 DPI. (G) Quantification of tissue-associated *C. rodentium* CFUs is shown.

(Figures 5F and 5G). Taken together, these results demonstrate that *C. rodentium* virulence gene expression alone is not sufficient for effective colonic mucosal colonization and that it is reliant on members of the gut microbiota for a physiological infection course.

DISCUSSION

Commensal bacteria are one of the first barriers of defense against invading pathogens at mucosal surfaces (Kamada et al., 2013). In this study, we investigated the role of commensals during the peak of enteric infection. We used the natural mouse pathogen *C. rodentium*, which can effectively colonize and trigger A/E lesions on the colonic mucosa, without antibiotic pre-treatment and in the presence of the endogenous microbiota. We show that antibiotic-induced dysbiosis with daily treatments of Kan gives rise to an avirulent population that persists in the lumen of the cecum and severely depletes the tissue-associated colonic population.

Pre-treatment of mice with antibiotics has been used to facilitate colonization of the mouse gut with *S*. Typhimurium (Barthel et al., 2003) and EHEC (Wadolkowski et al., 1990); however, this leads to habitation of the cecal lumen. We confirmed that, in the absence of the microbiota (i.e., in germ-free mice), *C. rodentium* mainly inhabits the cecal lumen, persisting at high titers avirulently (Kamada et al., 2012). We show that a persistent avirulent cecal population can also be induced with Kan treatment. Following humoral immunity-mediated eradication of virulent bacteria, commensal Proteobacteria are able to compete with *C. rodentium* for monosaccharides, effectively eliminating avirulent luminal *C. rodentium* (Kamada et al., 2012). Interestingly, we found Kan treatment results in a significant overgrowth of Bacteroidetes, primarily the *Bacteroides* and *Parabacteroides* genera. Members of the Bacteroidetes phylum, such as *B. thetaiotaomicron* and *Bacteroides vulgatus*, which can utilize both mono- and poly-saccharides, are ineffective at out-competing avirulent *C. rodentium* (Kamada et al., 2012). Together, this may explain the ability of *C. rodentium* to persist in mice following Kan treatment.

C. rodentium infection is associated with overgrowth of Enterobacteriaceae (Lupp et al., 2007), and we have recently shown that, during the peak of *C. rodentium* infection, elevated levels of gut cholesterol and mucosal O_2 cause a bloom in mucosal-associated γ-proteobacteria (Berger et al. 2017). Here, we show that the presence of gut commensals is required for colonization of the colonic mucosa; disruption of the microbiota with Kan leads to the rapid displacement of colonic tissue-associated *C. rodentium*. This phenomenon occurs when the microbiota is disrupted following intimate *C. rodentium* attachment and colonic A/E lesion formation. Met and Van treatment did not displace *C. rodentium* after four daily treatments; indeed, Met treatment has previously been found to exacerbate *C. rodentium*-induced colitis as a result of increased microbiotal degradation of the protective mucus layer (Wlodarska et al., 2011). Therefore, specific changes to the microbiome, induced by some antibiotic formulations, but not others, rather than a general reduction in diversity, displaces colonic *C. rodentium*.

Virulence of A/E pathogens is dependent on expression of LEE genes, which is regulated by a plethora of external stimuli, including microbiota-derived metabolites. Short-chain fatty acids (SCFAs), including succinate and butyrate, and mucin O-glycans, derived from degradation of gut mucans by commensals, such as *B. thetaiotaomicron*, all serve as regulators of the EHEC and *C. rodentium* T3SS (Curtis et al., 2014; Nakanishi et al., 2009; Pacheco et al., 2012). However, interestingly, similar to wild-type *C. rodentium*, we observed displacement of *C. rodentium* constitutively expressing *ler* from the colon following a single Kan treatment, demonstrating that this phenomenon occurs via a mechanism independent of Ler modulation. Therefore, our data show that, under conditions of specific dysbiosis, expression of virulence factors enabling A/E lesion formation is not sufficient for efficient colonization of the colonic mucosa. In germ-free mice, we observed a dramatic increase in *ler* gene expression between 2 and 8 DPI, which then decreased by 14 DPI, likely due to the regulatory feedback loop in which Ler inhibits its own transcription (Berdichevsky et al., 2005). This further demonstrates that the inability of *C. rodentium* to colonize the cecal and colonic mucosa in germ-free mice 8 DPI, when Ler is highly expressed, is not due to repression of virulence genes. Moreover, these findings suggest that host cues alone are able to control *ler* gene induction in *C. rodentium* colonizing germ-free mice.

Our data suggest that, during the peak of infection, rather than acting as a barrier for colonization, the gut microbiota are to some extent necessary to maintain colonic tissue-associated *C. rodentium* and, by extension, other enteric pathogens (e.g., EHEC and EPEC) via a mechanism independent of virulence gene modulation. Enteric pathogens, such as *Clostridium difficile* and *S.* Typhimurium, are reported to utilize microbiota-derived energy sources, including salic acid and fucose (Ng et al., 2013). *S.* Typhimurium exploits tetrathionate, a product of the reaction of reactive oxygen species (ROS) with microbiota-produced hydrogen sulfide as an alternative electron acceptor (Winter et al., 2010). It is interesting to note that the increase in gut cholesterol at 8 DPI is accompanied by a high level of cholesterol-metabolizing Proteobacteria, including *Serratia*, *Dickeya*, and *Erwinia* (Berger et al., 2017). *C. rodentium* is unable to grow on cholesterol as the sole carbon source; however, it is capable of utilizing degradation products, such as succinate (unpublished data), and it is interesting to speculate that *C. rodentium* may rely on other members of the microbiota to provide an energy source at the nutrient-poor epithelium. Moreover, depletion of butyrate-producing commensals by broad-spectrum antibiotics alters intestinal epithelial cell metabolism, resulting in increased luminal O_2 (Kelly et al., 2015; Rivera-Chávez et al., 2016). This would be expected to be beneficial to *C. rodentium*, which relies on aerobic respiration at the gut mucosa (Lopez et al., 2016). Indeed, we have recently shown that, during *C. rodentium* infection, the effector Map disrupts the mitochondrial function of intestinal epithelial cells to increase mucosal oxygenation (Berger et al., 2017). However, it is possible that an "over" saturation of O_2 in the gut mucosa (as a combined result of infection and antibiotic treatment) may be detrimental to *C. rodentium*, for example, by facilitating enhanced ROS production.

The fact that enteric pathogens may rely on commensals for colonization of mucosal surfaces emphasizes the importance of studying infection and pathogen–microbiome–host interactions at physiological anatomic sites in the gut within the context of the endogenous microbiota. Further work is required to fully elucidate the exact timescale and nature of dysbiosis induced following a single Kan treatment and the mechanism by which this excludes *C. rodentium* from the colonic mucosa.

EXPERIMENTAL PROCEDURES

Bacterial Strain Construction and Infections

C. rodentium strains were constructed by standard cloning methods. Western blot verification of strain phenotypes and cell culture infections were performed using standard techniques; see Supplemental Experimental Procedures and Tables S1 and S2.

Treatment of Mice with Antibiotics and Infection Studies

All animal experiments complied with the Animals Scientific Procedures Act 1986 and UK Home Office guidelines and were approved by the local ethical review committee. Experiments were designed in agreement with the ARRIVE guidelines (Kilkenny et al., 2010) for the reporting and execution of animal experiments, including sample randomization and blinding. Mouse experiments were performed with 3–8 mice/group and repeated on at least two separate occasions, with the exception of Figures S1 and 4A, which were performed once. Pathogen-free female 18–20 g C57BL/6 mice (Charles River Laboratories) were housed in high-efficiency particulate air (HEPA)-filtered cages with sterile bedding and given food and water *ad libitum*. Mice were infected with *C. rodentium* by oral gavage as described (Crepin et al., 2016). From 6 DPI, mice were gavaged with Cip (100 mg/kg/day in water), Kan (250–1,000 mg/kg/day in water), Van (50 mg/kg/day in water), Met (100 mg/kg/day in 5 mM HCl), or sterile water. For microbiome sequencing, naive, uninfected mice were gavaged with antibiotics; stools were collected prior to treatment and 24 hr after 3 daily treatments and immediately flash frozen.

Germ-free Mice

All germ-free animal studies were approved by the Weizmann Institute of Science Institutional Animal Care and Use Committee (IACUC), application number 28050616. Germ-free mice were born in the Weizmann Institute germ-free facility and routinely monitored for sterility; on the day of inoculation, germ-free mice were transferred into sterile iso-cages (Hecht et al., 2014). Germ-free mice were given a regular chow diet *ad libitum* and infected with *C. rodentium* as described above.

Co-housing Experiments

An AIBP donor mouse (26 DPI with ICC180; post-20 Kan 1,000 mg/kg/day treatments) was co-housed with naive, untreated recipient mice. As a control, an untreated, ICC180-infected mouse (7 DPI) was separately co-housed with naive mice. In both cases, the donor mice were removed after 72 hr.

In Vivo Optical Imaging of *C. rodentium*-Infected Mice

Whole-animal bioluminescence imaging (BLI) was performed using an IVIS Spectrum CT (PerkinElmer; Crepin et al., 2016). At necropsy on the days indicated, excised gastrointestinal tissues with the mucosa exteriorized were also imaged.

Sample Processing and Histological Analysis

Colonization was monitored by enumeration of viable bacteria (CFU) per gram of feces or tissue as indicated. Histological analysis (CCH measurements) and immuno-staining were performed as described (Crepin et al., 2016); see Supplemental Experimental Procedures.

Measurement of Gut Transit Times

Mice were given a single antibiotic dose, as described above. 24 hr later, mice were gavaged with 300 μL 6% (w/v) carmine red in 0.5% (w/v) methyl cellulose solution. Mice were housed in individual cages and given food and water *ad libitum.* Total gut transit was recorded as the time from gavage until the passage of the first red stool rounded up to the nearest 10 min.

LCN-2 Measurement

Stool samples were homogenized in PBS + 0.1% Tween-20. Samples were centrifuged at 16,000 rpm for 10 min and the supernatant extracted and stored at −80°C. LCN-2 concentration was determined using a DuoSet Mouse Lipocalin-2/NGAL ELISA (R&D Systems), according to the manufacturer's instructions.

16S Sequencing and Analysis

Stool samples were processed for DNA isolation using MoBio (PowerSoil kit) according to the manufacturer's instructions. The purified DNA from feces was used for PCR amplification and sequencing of the bacterial 16S rRNA gene. Amplicons of ~380 base pairs spanning the variable region 3 or 4 (V3–4) of the 16S rRNA gene were generated by using designated primers. The PCR products were subsequently pooled in an equimolar ratio, purified (PCR clean kit; Promega), and used for Illumina MiSeq sequencing. Reads were processed using the QIIME (quantitative insights into microbial ecology) analysis pipeline as previously described (Elinav et al., 2011) version 1.8. Paired-end joined sequences were grouped into operational taxonomic units (OTUs) using the UCLUST algorithm and the GreenGenes database (DeSantis et al., 2006). Sequences with distance-based similarity of 97% or greater over median sequence length of 353 base pairs were assigned to the same OTU. Analysis was performed at each taxonomical level (phylum to genus and species level if possible) separately. For each taxon, statistical tests were performed between the different groups. p values were false discovery rate (FDR) corrected for multiple hypothesis testing.

Statistical Analyses

Data were analyzed by a paired or unpaired two-tailed Student's t test or one-way ANOVA with Tukey's multiple comparison post-test, as specified in figure legends. A commercially available software (GraphPad 7) was used; a p value of <0.05 was taken to be significant.

SUPPLEMENTAL INFORMATION

Supplemental Information includes Supplemental Experimental Procedures, two figures, and two tables and can be found with this article online at https://doi.org/10.1016/j.celrep.2017.11.086.

ACKNOWLEDGMENTS

E.E. is supported by Y. and R. Ungar, the Leona M. and Harry B. Helmsley Charitable Trust, and grants funded by the European Research Council and is a senior fellow at the Canadian Institute for Advanced Research (CIFAR) and an International Scholar at the Bill and Melinda Gates Foundation and Howard Hughes Medical Institute (HHMI). This work was supported by grant MR/N00695X/1 from the AMR cross research council initiative under theme 1, understanding resistant bacteria in the context of the host (C.M.-S. and G.F.), and a Royal Society International Collaboration Award for Research Professors, ref: IC160080 (G.F.).

AUTHOR CONTRIBUTIONS

C.M.-S., J.W.C., I.T.G.-M., A.M.S., J.L.C.W., and V.F.C. conducted the *in vivo* studies. D.R.-G. constructed *C. rodentium* strains. M.L. conducted the germ-free *in vivo* studies. M.P.-F. profiled the microbiota. E.E. and G.F. provided supervision and guidance. A.M.S. illustrated the graphical abstract. C.M.-S., J.W.C., and G.F. wrote the paper.

DECLARATION OF INTERESTS

The authors declare no competing interests.

Received: July 22, 2017
Revised: September 25, 2017
Accepted: November 27, 2017
Published: December 19, 2017

REFERENCES

Barthel, M., Hapfelmeier, S., Quintanilla-Martínez, L., Kremer, M., Rohde, M., Hogardt, M., Pfeffer, K., Rüssmann, H., and Hardt, W.-D. (2003). Pretreatment of mice with streptomycin provides a *Salmonella enterica* serovar Typhimurium colitis model that allows analysis of both pathogen and host. Infect. Immun. *71*, 2839–2858.

Berdichevsky, T., Friedberg, D., Nadler, C., Rokney, A., Oppenheim, A., and Rosenshine, I. (2005). Ler is a negative autoregulator of the LEE1 operon in enteropathogenic *Escherichia coli*. J. Bacteriol. *187*, 349–357.

Berger, C.N., Crepin, V.F., Roumeliotis, T.I., Wright, J.C., Carson, D., Pevsner-Fischer, M., Furniss, R.C.D., Dougan, G., Dori-Bachash, M., Yu, L., et al. (2017). *Citrobacter rodentium* subverts ATP flux and cholesterol homeostasis in intestinal epithelial cells *in vivo*. Cell Metab. *26*, 738–752.e6.

Collins, J.W., Meganck, J.A., Kuo, C., Francis, K.P., and Frankel, G. (2013). 4D multimodality imaging of *Citrobacter rodentium* infections in mice. J. Vis. Exp. *78*, e50450.

Collins, J.W., Keeney, K.M., Crepin, V.F., Rathinam, V.A., Fitzgerald, K.A., Finlay, B.B., and Frankel, G. (2014). *Citrobacter rodentium*: infection, inflammation and the microbiota. Nat. Rev. Microbiol. *12*, 612–623.

Crepin, V.F., Girard, F., Schüller, S., Phillips, A.D., Mousnier, A., and Frankel, G. (2010). Dissecting the role of the Tir:Nck and Tir:IRTKS/IRSp53 signalling pathways *in vivo*. Mol. Microbiol. *75*, 308–323.

Crepin, V.F., Collins, J.W., Habibzay, M., and Frankel, G. (2016). *Citrobacter rodentium* mouse model of bacterial infection. Nat. Protoc. *11*, 1851–1876.

Curtis, M.M., Hu, Z., Klimko, C., Narayanan, S., Deberardinis, R., and Sperandio, V. (2014). The gut commensal *Bacteroides thetaiotaomicron* exacerbates enteric infection through modification of the metabolic landscape. Cell Host Microbe *16*, 759–769.

Deng, W., Puente, J.L., Gruenheid, S., Li, Y., Vallance, B.A., Vázquez, A., Barba, J., Ibarra, J.A., O'Donnell, P., Metalnikov, P., et al. (2004). Dissecting virulence: systematic and functional analyses of a pathogenicity island. Proc. Natl. Acad. Sci. USA *101*, 3597–3602.

DeSantis, T.Z., Hugenholtz, P., Larsen, N., Rojas, M., Brodie, E.L., Keller, K., Huber, T., Dalevi, D., Hu, P., and Andersen, G.L. (2006). Greengenes, a chimera-checked 16S rRNA gene database and workbench compatible with ARB. Appl. Environ. Microbiol. *72*, 5069–5072.

Elinav, E., Strowig, T., Kau, A.L., Henao-Mejia, J., Thaiss, C.A., Booth, C.J., Peaper, D.R., Bertin, J., Eisenbarth, S.C., Gordon, J.I., and Flavell, R.A.

(2011). NLRP6 inflammasome regulates colonic microbial ecology and risk for colitis. Cell *145*, 745–757.

Garmendia, J., Frankel, G., and Crepin, V.F. (2005). Enteropathogenic and enterohemorrhagic *Escherichia coli* infections: translocation, translocation, translocation. Infect. Immun. *73*, 2573–2585.

Ghosh, S., Dai, C., Brown, K., Rajendiran, E., Makarenko, S., Baker, J., Ma, C., Halder, S., Montero, M., Ionescu, V.-A., et al. (2011). Colonic microbiota alters host susceptibility to infectious colitis by modulating inflammation, redox status, and ion transporter gene expression. Am. J. Physiol. Gastrointest. Liver Physiol. *301*, G39–G49.

Hecht, G., Bar-Nathan, C., Milite, G., Alon, I., Moshe, Y., Greenfeld, L., Dotsenko, N., Suez, J., Levy, M., Thaiss, C.A., et al. (2014). A simple cage-autonomous method for the maintenance of the barrier status of germ-free mice during experimentation. Lab. Anim. *48*, 292–297.

Islam, M.S., Bingle, L.E., Pallen, M.J., and Busby, S.J. (2011). Organization of the LEE1 operon regulatory region of enterohaemorrhagic *Escherichia coli* O157:H7 and activation by GrlA. Mol. Microbiol. *79*, 468–483.

Kamada, N., Kim, Y.-G., Sham, H.P., Vallance, B.A., Puente, J.L., Martens, E.C., and Núñez, G. (2012). Regulated virulence controls the ability of a pathogen to compete with the gut microbiota. Science *336*, 1325–1329.

Kamada, N., Chen, G.Y., Inohara, N., and Núñez, G. (2013). Control of pathogens and pathobionts by the gut microbiota. Nat. Immunol. *14*, 685–690.

Kelly, C.J., Zheng, L., Campbell, E.L., Saeedi, B., Scholz, C.C., Bayless, A.J., Wilson, K.E., Glover, L.E., Kominsky, D.J., Magnuson, A., et al. (2015). Cross-talk between microbiota-derived short-chain fatty acids and intestinal epithelial HIF augments tissue barrier function. Cell Host Microbe *17*, 662–671.

Kilkenny, C., Browne, W.J., Cuthill, I.C., Emerson, M., and Altman, D.G. (2010). Improving bioscience research reporting: the ARRIVE guidelines for reporting animal research. PLoS Biol. *8*, e1000412.

Lio, J.C.-W., and Syu, W.-J. (2004). Identification of a negative regulator for the pathogenicity island of enterohemorrhagic *Escherichia coli* O157:H7. J. Biomed. Sci. *11*, 855–863.

Lopez, C.A., Miller, B.M., Rivera-Chávez, F., Velazquez, E.M., Byndloss, M.X., Chávez-Arroyo, A., Lokken, K.L., Tsolis, R.M., Winter, S.E., and Bäumler, A.J. (2016). Virulence factors enhance *Citrobacter rodentium* expansion through aerobic respiration. Science *353*, 1249–1253.

Lupp, C., Robertson, M.L., Wickham, M.E., Sekirov, I., Champion, O.L., Gaynor, E.C., and Finlay, B.B. (2007). Host-mediated inflammation disrupts the intestinal microbiota and promotes the overgrowth of Enterobacteriaceae. Cell Host Microbe *2*, 119–129.

McDaniel, T.K., Jarvis, K.G., Donnenberg, M.S., and Kaper, J.B. (1995). A genetic locus of enterocyte effacement conserved among diverse enterobacterial pathogens. Proc. Natl. Acad. Sci. USA *92*, 1664–1668.

Mellies, J.L., Elliott, S.J., Sperandio, V., Donnenberg, M.S., and Kaper, J.B. (1999). The Per regulon of enteropathogenic *Escherichia coli* : identification of a regulatory cascade and a novel transcriptional activator, the locus of enterocyte effacement (LEE)-encoded regulator (Ler). Mol. Microbiol. *33*, 296–306.

Nakanishi, N., Tashiro, K., Kuhara, S., Hayashi, T., Sugimoto, N., and Tobe, T. (2009). Regulation of virulence by butyrate sensing in enterohaemorrhagic *Escherichia coli*. Microbiology *155*, 521–530.

Ng, K.M., Ferreyra, J.A., Higginbottom, S.K., Lynch, J.B., Kashyap, P.C., Gopinath, S., Naidu, N., Choudhury, B., Weimer, B.C., Monack, D.M., and Sonnenburg, J.L. (2013). Microbiota-liberated host sugars facilitate post-antibiotic expansion of enteric pathogens. Nature *502*, 96–99.

Pacheco, A.R., Curtis, M.M., Ritchie, J.M., Munera, D., Waldor, M.K., Moreira, C.G., and Sperandio, V. (2012). Fucose sensing regulates bacterial intestinal colonization. Nature *492*, 113–117.

Rivera-Chávez, F., Zhang, L.F., Faber, F., Lopez, C.A., Byndloss, M.X., Olsan, E.E., Xu, G., Velazquez, E.M., Lebrilla, C.B., Winter, S.E., and Bäumler, A.J. (2016). Depletion of butyrate-producing *Clostridia* from the gut microbiota drives an aerobic luminal expansion of *Salmonella*. Cell Host Microbe *19*, 443–454.

Spees, A.M., Wangdi, T., Lopez, C.A., Kingsbury, D.D., Xavier, M.N., Winter, S.E., Tsolis, R.M., and Bäumler, A.J. (2013). Streptomycin-induced inflammation enhances *Escherichia coli* gut colonization through nitrate respiration. MBio *4*, e00430–13.

Wadolkowski, E.A., Burris, J.A., and O'Brien, A.D. (1990). Mouse model for colonization and disease caused by enterohemorrhagic *Escherichia coli* O157:H7. Infect. Immun. *58*, 2438–2445.

Wiles, S., Clare, S., Harker, J., Huett, A., Young, D., Dougan, G., and Frankel, G. (2004). Organ specificity, colonization and clearance dynamics *in vivo* following oral challenges with the murine pathogen *Citrobacter rodentium*. Cell. Microbiol. *6*, 963–972.

Winter, S.E., Thiennimitr, P., Winter, M.G., Butler, B.P., Huseby, D.L., Crawford, R.W., Russell, J.M., Bevins, C.L., Adams, L.G., Tsolis, R.M., et al. (2010). Gut inflammation provides a respiratory electron acceptor for *Salmonella*. Nature *467*, 426–429.

Wlodarska, M., Willing, B., Keeney, K.M., Menendez, A., Bergstrom, K.S., Gill, N., Russell, S.L., Vallance, B.A., and Finlay, B.B. (2011). Antibiotic treatment alters the colonic mucus layer and predisposes the host to exacerbated *Citrobacter rodentium*-induced colitis. Infect. Immun. *79*, 1536–1545.

Wong, A.R., Pearson, J.S., Bright, M.D., Munera, D., Robinson, K.S., Lee, S.F., Frankel, G., and Hartland, E.L. (2011). Enteropathogenic and enterohaemorrhagic *Escherichia coli*: even more subversive elements. Mol. Microbiol. *80*, 1420–1438.

Wostmann, B., and Bruckner-Kardoss, E. (1960). Cecal enlargement in germ-free animals. Nutr. Rev. *18*, 313–314.

Zhang, L., Huang, Y., Zhou, Y., Buckley, T., and Wang, H.H. (2013). Antibiotic administration routes significantly influence the levels of antibiotic resistance in gut microbiota. Antimicrob. Agents Chemother. *57*, 3659–3666.

Cell Reports, Volume *21*

Supplemental Information

Citrobacter rodentium Relies on Commensals for Colonization of the Colonic Mucosa

Caroline Mullineaux-Sanders, James W. Collins, David Ruano-Gallego, Maayan Levy, Meirav Pevsner-Fischer, Izabela T. Glegola-Madejska, Agnes M. Sågfors, Joshua L.C. Wong, Eran Elinav, Valerie F. Crepin, and Gad Frankel

Supplemental Figures

A

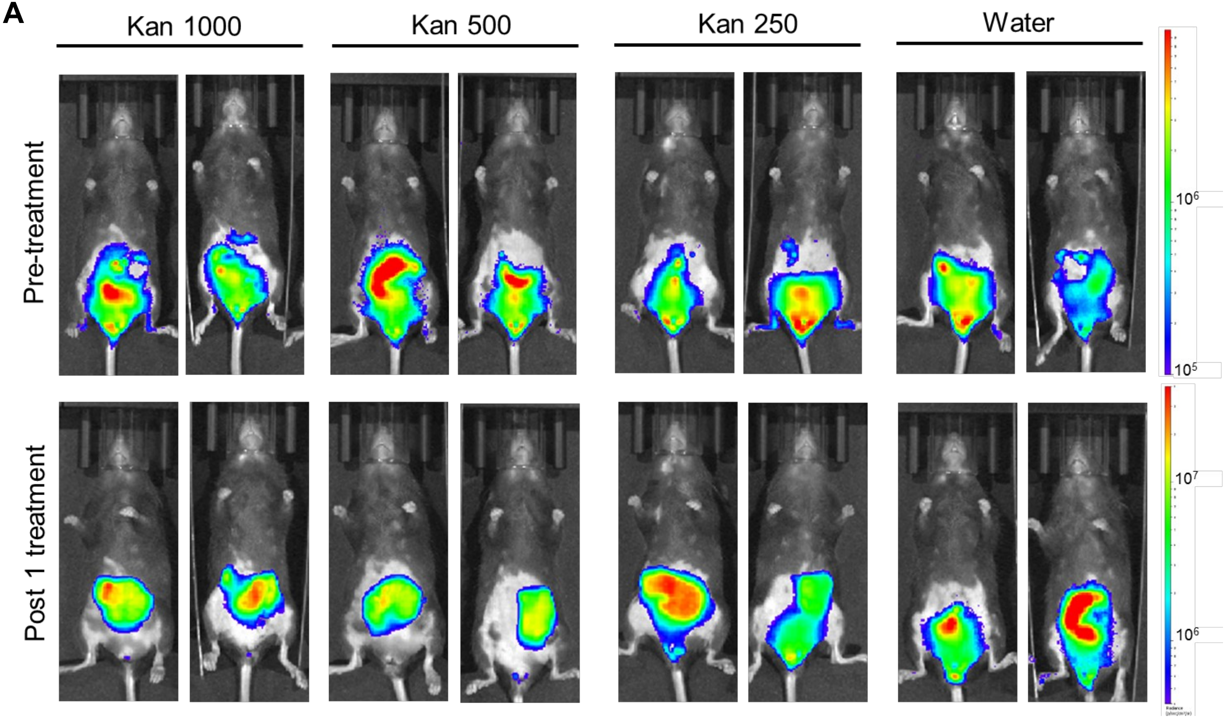

B

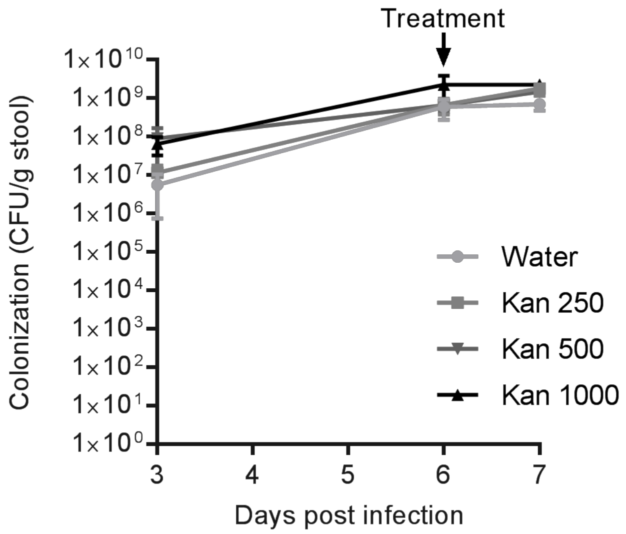

Figure S1. Characterization of the dose-dependent response to Kan treatment; related to Fig. 1. A) *In vivo* BLI of *C. rodentium* from mice before, and 24 h following, a single treatment with 1000 mg/kg, 500 mg/kg, 250 mg/kg Kan or a water control, as indicated. **B)** Quantification of *C. rodentium* CFUs in stools of mice treated as for Fig. S1A. Mean values ± SEM, n=5 mice.

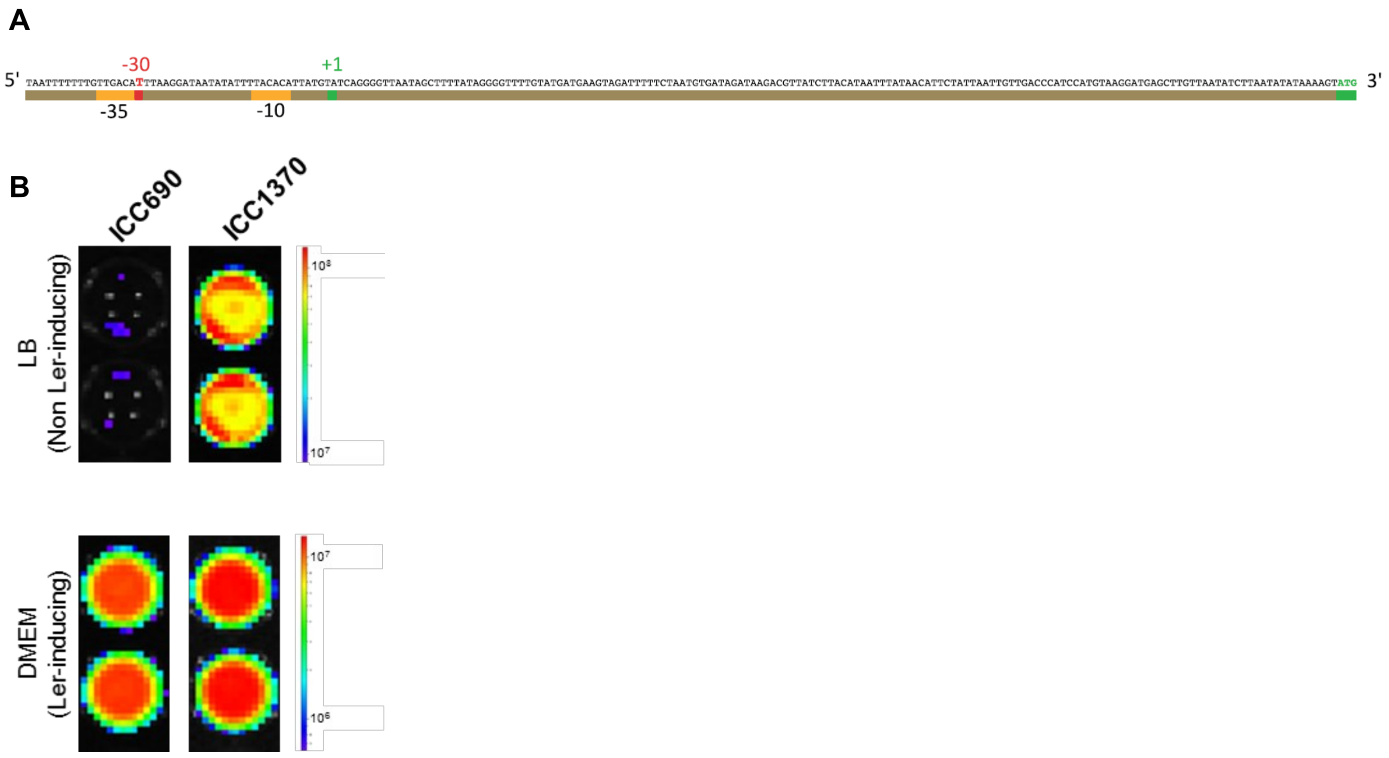

Figure S2. ICC690 and ICC1370; related to Fig. 2C-D and Fig. 5F. A) Sequence of the *ler* promoter; +1=start of transcription; ATG=start of translation. Deletion of the base pair at -30 (shown in red) renders the promoter constitutive. **B)** ICC690 is non-BL when grown under non Ler-inducing conditions (LB); ICC1370 remains BL; ICC690 and ICC1370 are BL when grown under Ler-inducing conditions (DMEM).

Table S1. Strains and Plasmids used in this study; related to Experimental Procedures.

Name	Description	Reference
**Strains**		
E. coli CC118-λpir	Δ(*ara-leu*) *araD* Δ*lacX74 galE galK phoA20 thi- rpsE rpoB argE(Am) recA1, λpir*	De Lorenzo *et al.*, 1990
E. coli 1047 pRK2013	Helper strain for conjugation, KanR, *oriColE1 RK2-Mob+ RK2-Tra+*	Figurski and Helinski, 1979
ICC169	Wild-type *C. rodentium*, O152 serotype, NalR	Wiles *et al.*, 2004
ICC180	Luminescent ICC169 derivative, NalR, KanR	Wiles *et al.*, 2004
ICC690	*C. rodentium* Pler-dependent luminescent strain, NalR, KanR	This study
ICC1370	*C. rodentium* constitutive-Pler luminescent strain, NalR, KanR	This study
ICC1410	ICC180 derivative, Δ*grlR*::CmR; NalR, KanR	This study
**Plasmids**		
pGEmatP2lux	Plasmid harboring the *luxCDABE* operon of *Photorabdus luminescens*	Piñero-Lambea *et al.*, 2014
pACYC184-XylE	pACYC184 derivate; *xylE* homology regions of *C. rodentium*	Girard *et al.*, 2009
pACYC184-XylE-Pler-lux	pACYC184-XylE derivative; *lux* operon under the *C. rodentium ler* promoter	This study
pACYC184-XylE-Pler*-lux	pACYC184-XylE derivative; *lux* operon under *C. rodentium* constitutive *ler* promoter	This study
pICC618	Plasmid encoding the chloramphenicol resistance *cat* cassette	Wong *et al.*, 2012
pSEVA612S	GmR; R6K ori, oriT, I-SceI restriction sites flanking multicloning site	Martínez-García *et al.*, 2014
pSEVAΔ*grlR*-Cm	pSEVA612S derivative; *grlR* homology regions of *C. rodentium* flanking the *cat* cassette	This study
pACBSR	SpR/SmR; p15A ori, PBAD, I-SceI endonuclease and λ-red genes	Ruano-Gallego *et al.*, 2015

Table S2. Primers used in this study; related to Experimental Procedures.

Name	Sequence (5'-3')
5-XbaI-Pler	TAATCTAGAGCTTTGCCAACTAGCTAAATC
3-HindIII-Pler	GACAAGCTTACTTTTATATATTAAGATATTAACAAGC
5-HindIII-lux	AATAGATTCATTCTAGAAAGC
3-SphI-lux	ATTAGCATGCTCAACTATCAAACGCTTCGGTTAAG
5-pler*-GB2	TGATACATAATGTGTAAAATATATTATCCTTAATGTCAACAAAAAAATTAT ACC
3-pler*-GB2	ACATTAAGGATAATATATTTTACACATTATGTATCAGGGGTTAATAGCTTTT ATAG
5-HR-XylE-UP	CTATTGCAGCAACCGCTTTGGCC
3-HR-XylE-DO	AAGTAGTTTGCCAGCCACTGGGC
XylE-UP-gen-check	CAAAATTTAAGTGAATCCGCCGCCAACTCG
3-luxC-seq	TGGGACAAATACAAGGAACTTATCTTCTTC
grlR-gen-check-UP	TTATTTGCATTATCCCTATATCATAGGTTCC
grlR-gen-check-DO	GTCCCACAATACCATTACCAACTCGTAAGG
5-SalI-HR-GrlR	ATCAGTCGACATGGAATCTAAAAATAGTGAC
3-SalI-HR-GrlR	ATCAGTCGACTTTCATGCTAACCTCACTCC

Supplemental Experimental Procedures

Bacterial Strains. The bacterial strains and plasmids used in this study are listed in Table S1. Bacteria were grown at 37°C on Lysogeny broth (LB) agar (Merck) plates (1.5% w/v), in liquid LB medium (Merck), Dulbecco's modified Eagle's medium (DMEM, Sigma), or M9 minimal medium. When required, antibiotics were added at the following concentrations: chloramphenicol (Cm) 30 μg/ml; kanamycin (Kan) 50 μg/ml; gentamicin (Gm) 10 μg/ml; streptomycin (Sm) 50 μg/ml, nalidixic acid (Nal) 50 μg/ml. We confirmed that ICC180 is sensitive to ciprofloxacin (Cip; <0.25 μg/ml) and resistant to Kan, metronidazole (Met) and vancomycin (Van) (>128 μg/ml).

Lamda Red chromosomal mutations (ICC690 and ICC1370). Primers (Sigma) used in this study are listed in Table S2. The *ler* promoter (Pler) was amplified from *C. rodentium* genomic DNA using primers 5-XbaI-Pler and 3-HindIII-Pler and cloned into pACYC184-XylE. The *lux* operon from *Photorhabdus luminescens* was amplified by PCR from pGEmatP2lux (Piñero-Lambea *et al.*, 2014) with primers 5-HindIII-lux and 3-SphI-lux and inserted in the pCR-BluntII-TOPO plasmid (Zero Blunt TOPO PCR Cloning Kit, Life Technologies). The *lux* operon was subsequently inserted into pACYC184-XylE-Pler to obtain pACYC184-XylE-Pler-lux. To perform the point mutation in the *ler* promoter (Pler*), pACYC184-XylE-Pler-lux was reverse amplified with primers 5-pler*-GB2 and 3-pler*-GB2 and ligated using a Gibson assembly kit (New England Biolabs). To obtain linear products for transformation into ICC169, plasmids pACYC184-XylE-Pler-lux and pACYC184-XylE-Pler*-lux were reverse amplified by PCR using primers 5-HR-XylE-UP and 3-HR-XylE-DO. The PCR products were transformed into electrocompetent ICC169 harbouring pACBSR, previously induced with 0.4% (w/v) L-arabinose (Sigma). Bacteria were incubated for 4 h at 37°C and Cm-resistant conjugants selected. Integration at the correct genomic location was confirmed by PCR analysis and sequencing using primers XylE-UP-gen-check and 3-luxC-seq. After several passages in liquid LB to remove pACBSR from the strain, bacteria sensitive to Sm were selected.

pSEVA chromosomal mutations (ICC1410). Homology regions flanking the *grlR* gene were synthesized (GenArt, Thermo) and cloned into the suicide vector pSEVA612S (Martínez-García *et al.*, 2014). The construct was then reverse amplified using primers 5-SalI-HR-GrlR and 3-SalI-HR-GrlR and ligated with a Cm resistance cassette digested from pICC618 using SalI. The resulting plasmid pSEVAΔ*grlR*-Cm was conjugated into *C. rodentium* ICC180. Briefly, 20 µl helper *E. coli* 1047 pRK2013 was incubated for 2h at 37°C with 20 µl of the donor strain (*E. coli* CC118-λpir pSEVAΔ*grlR*) on LB agar. Next, 40 µl of the receiver strain (*C. rodentium* ICC180 with pACBSR) was added and the plate incubated for 4 h at 37°C. Conjugants were selected on LB

agar supplemented with Gm and Sm. Individual colonies were grown in LB supplemented with Sm and 0.4% (w/v) L-arabinose (Sigma) for 8 h to induce expression of the I-SceI endonuclease from pACBSR, and plated on Cm plates. Genomic deletion of Δ*grlR* was verified by PCR using primers grlR-gen-check-UP and grlR-gen-check-DO. The strains were passaged several times in liquid LB to remove pACBSR and bacteria sensitive to Sm were selected.

T3SS secretion assay. For the analysis of the T3 secreted proteins, ICC1410 and ICC169 were grown in LB at 37°C, 200 rpm (non-LEE inducing conditions). To analyze the secretion of T3SS components, culture supernatants were isolated from 1 ml aliquots of induced cultures by centrifugation (20000 g, 5 min). Supernatants were subsequently chilled on ice and incubated for 60 min with trichloroacetic acid (TCA 20% w/v; Sigma) for precipitation. After cold centrifugation (20000 g, 15 min), TCA-precipitated protein pellets were rinsed with cold acetone (-20°C) and resuspended in 30 µl of Sodium Dodecyl Sulfate–Polyacrylamide gel electrophoresis (SDS-PAGE) sample buffer.

Western blots. SDS-PAGE and Western blot were performed following standard methods using the Miniprotean III system (Bio-Rad). Proteins separated by SDS-PAGE were either subjected to Coomassie Blue R-250 (Sigma) staining or Western blot. For the latter, the proteins were transferred to a polyvinylidene difluoride membrane (PVDF, GE Healthcare) using semi-dry electrophoresis (Bio-Rad). Membranes were incubated with rabbit anti-EspA (1:5000) or anti-EspB (1:5000) antibodies followed by secondary goat anti-rabbit antibody conjugated to HRP (1:500, Jackson ImmunoResearch). Membranes were developed by chemiluminescence using the EZ-ECL kit (Biological Industries) and observed in a Fuji LAS 3000 Imager (Fujifilm).

Cell culture and infection. Swiss 3T3 and HeLa (ATCC) cell lines were cultured and seeded onto glass coverslips in 24-well plates and infected with *C. rodentium* as previously described (Girard *et al.*, 2009). For ICC180 and ICC1410 infections, cultures grown for 8 hours in LB (37°C, 200 rpm) were diluted 1/100 in DMEM and grown overnight at 37°C, 5% CO_2. 100 µl of overnight culture was added to each well. Plates were incubated at 37°C, 5% CO_2 for 3 hours. Cells were washed in PBS and fixed for 20 min in 4% paraformaldehyde (Collins *et al.*, 2014b). For infections with *C. rodentium* isolated from infected mice, fresh fecal samples taken from donor mice were diluted 1/10 in PBS, homogenized and then 20 µl of the supernatant was immediately added to the wells (Bishop *et al.*, 2007). Control wells were infected with 100 µl of an overnight culture, prepared as described above. Plates were centrifuged at 1000 rpm for 5 min at room temperature, and were incubated at 37°C in 5% CO_2 for 5 h, then washed and fixed as described above.

Colonic crypt measurements. 0.5 cm distal colon was harvested, fixed in formalin, sectioned and stained with hematoxylin and eosin as previously described (Crepin *et al.,* 2016). Crypt hyperplasia was determined by measuring the lengths of at least 20 well-oriented crypts from each section, from all of the mice per treatment group. All histological sections were evaluated blindly.

Indirect immunofluorescence staining. Fixed cells were permeabilized in 0.1% Triton X100 (Sigma) for 10 min at room temperature. For tissue sections, indirect immunofluorescence was performed following heat induced epitope retrieval of formalin-fixed paraffin embedded sections as previously described (Collins *et al.*, 2014b). Samples were treated with primary rabbit polyclonal anti-*C. rodentium* (1:50 for tissue sections, 1:200 for fixed cells; Sigma) and mouse anti-E-Cadherin antibodies (1:50; CD324, BD Biosciences). Samples were subsequently treated with secondary antibodies: Donkey anti-rabbit Alexa Flour 488, donkey anti-mouse Alexa Flour 488 or donkey anti-rabbit Alexa Flour 555 (all 1:200; Jackson Immunoresearch). Actin was counter-stained with Phalloidin (Stratech) and DNA was counter-stained with Hoescht 33342 or DAPI. Images were acquired using a Zeiss AxioVision Z3 microscope, and processed using Zen 2.3 (Blue Version) (Carl Zeiss MicroImaging GmbH, Germany).

Supplemental References

Bishop, A.L., Wiles, S., Dougan, G., and Frankel, G. (2007). Cell attachment properties and infectivity of host-adapted and environmentally adapted *Citrobacter rodentium*. Microb. Infect. *9*, 1316-1324.

Collins, J.W., Chervaux, C., Raymond, B., Derrien, M., Brazeilles, R., Kosta, A., Chambaud, I., Crepin, V.F., and Frankel, G. (2014b). Fermented dairy products modulate *C. rodentium* induced colonic hyperplasia. J. Infect. Dis. *210*, 1029-1041.

De Lorenzo, V., Herrero, M., Jakubzik, U., and Timmis, K.N. (1990). Mini-Tn5 transposon derivatives for insertion mutagenesis, promoter probing, and chromosomal insertion of cloned DNA in gram-negative eubacteria. J. Bacteriol. *172*, 6568-6572.

Figurski, D.H., and Helinski, D.R. (1979). Replication of an origin-containing derivative of plasmid RK2 dependent on a plasmid function provided in trans. Proc. Natl. Acad. Sci. U.S.A. *76*, 1648-1652.

Girard, F., Crepin, V.F., and Frankel, G. (2009). Modelling of infection by enteropathogenic *Escherichia coli* strains in lineages 2 and 4 *ex vivo* and *in vivo* by using *Citrobacter rodentium* expressing TccP. Infect. Immun. *77*, 1304-1314.

Martínez-García, E., Aparicio, T., Goñi-Moreno, A., Fraile, S., and De Lorenzo, V. (2014). SEVA 2.0: an update of the Standard European Vector Architecture for de-/re-construction of bacterial functionalities. Nucleic Acids Res. *43,* D1183–D1189.

Piñero-Lambea, C., Bodelón, G., Fernández-Periáñez, R., Cuesta, A.M., Álvarez-Vallina, L., and Fernández, L.A.n. (2014). Programming controlled adhesion of *E. coli* to target surfaces, cells, and tumors with synthetic adhesins. ACS Synth. Biol. *4*, 463-473.

Ruano-Gallego, D., Álvarez, B., and Fernández, L.A.n. (2015). Engineering the controlled assembly of filamentous injectisomes in *E. coli* K-12 for protein translocation into mammalian cells. ACS Synth. Biol. *4*, 1030-1041.

Wong, A.R., Raymond, B., Collins, J.W., Crepin, V.F., and Frankel, G. (2012). The enteropathogenic *E. coli* effector EspH promotes actin pedestal formation and elongation via WASP‐interacting protein (WIP). Cell Microbiol. *14*, 1051-1070.
